# Supplementary material for: Threshold analysis regarding the optimal tax rate and tax evasion. Empirical evidence from Taiwan
Source: PLoS One. 2023 Mar 31;18(3):e0281101. doi: 10.1371/journal.pone.0281101 (PMC10065287; doi:10.1371/journal.pone.0281101)
Supplement: S1 File — (DOCX) [file pone.0281101.s001.docx]

| Dependent Variable: TAIWANGDP | | | |  |
| --- | --- | --- | --- | --- |
| Method: Least Squares | | | |  |
| Date: 08/11/21 Time: 11:56 | | | |  |
| Sample (adjusted): 1993 2020 | | | |  |
| Included observations: 28 after adjustments | | | | |
| Convergence achieved after 3 iterations | | | | |
|  |  |  |  |  |
|  |  |  |  |  |
| Variable | Coefficient | Std. Error | t-Statistic | Prob. |
|  |  |  |  |  |
|  |  |  |  |  |
| C | 12791933 | 37883804 | 0.337662 | 0.7384 |
| AR(1) | 1.716352 | 0.271115 | 6.330718 | 0.0000 |
| AR(2) | -0.720552 | 0.270556 | -2.663221 | 0.0133 |
|  |  |  |  |  |
|  |  |  |  |  |
| R-squared | 0.987818 | Mean dependent var | | 12788620 |
| Adjusted R-squared | 0.986844 | S.D. dependent var | | 3865710. |
| S.E. of regression | 443401.0 | Akaike info criterion | | 28.94329 |
| Sum squared resid | 4.92E+12 | Schwarz criterion | | 29.08603 |
| Log likelihood | -402.2061 | Hannan-Quinn criter. | | 28.98693 |
| F-statistic | 1013.622 | Durbin-Watson stat | | 2.461040 |
| Prob(F-statistic) | 0.000000 |  |  |  |
|  |  |  |  |  |
|  |  |  |  |  |
| Inverted AR Roots | .98 | .73 | | |
|  |  |  |  |  |
|  |  |  |  |  |

| Dependent Variable: TTR | | | |  |
| --- | --- | --- | --- | --- |
| Method: Least Squares | | | |  |
| Date: 08/11/21 Time: 11:58 | | | |  |
| Sample (adjusted): 1993 2020 | | | |  |
| Included observations: 28 after adjustments | | | | |
| Convergence achieved after 12 iterations | | | | |
|  |  |  |  |  |
|  |  |  |  |  |
| Variable | Coefficient | Std. Error | t-Statistic | Prob. |
|  |  |  |  |  |
|  |  |  |  |  |
| C | 2981412. | 2465239. | 1.209381 | 0.2378 |
| AR(1) | 0.613109 | 0.189005 | 3.243884 | 0.0033 |
| AR(2) | 0.334832 | 0.192494 | 1.739441 | 0.0943 |
|  |  |  |  |  |
|  |  |  |  |  |
| R-squared | 0.824595 | Mean dependent var | | 1669028. |
| Adjusted R-squared | 0.810562 | S.D. dependent var | | 424352.5 |
| S.E. of regression | 184697.0 | Akaike info criterion | | 27.19178 |
| Sum squared resid | 8.53E+11 | Schwarz criterion | | 27.33451 |
| Log likelihood | -377.6849 | Hannan-Quinn criter. | | 27.23541 |
| F-statistic | 58.76359 | Durbin-Watson stat | | 2.073831 |
| Prob(F-statistic) | 0.000000 |  |  |  |
|  |  |  |  |  |
|  |  |  |  |  |
| Inverted AR Roots | .96 | -.35 | | |
|  |  |  |  |  |
|  |  |  |  |  |

| Dependent Variable: DTR | | | |  |
| --- | --- | --- | --- | --- |
| Method: Least Squares | | | |  |
| Date: 08/11/21 Time: 12:01 | | | |  |
| Sample (adjusted): 1993 2020 | | | |  |
| Included observations: 28 after adjustments | | | | |
| Convergence achieved after 11 iterations | | | | |
|  |  |  |  |  |
|  |  |  |  |  |
| Variable | Coefficient | Std. Error | t-Statistic | Prob. |
|  |  |  |  |  |
|  |  |  |  |  |
| C | 1528792. | 1130838. | 1.351910 | 0.1885 |
| AR(1) | 0.783187 | 0.202701 | 3.863743 | 0.0007 |
| AR(2) | 0.161992 | 0.210410 | 0.769886 | 0.4486 |
|  |  |  |  |  |
|  |  |  |  |  |
| R-squared | 0.846134 | Mean dependent var | | 894309.1 |
| Adjusted R-squared | 0.833825 | S.D. dependent var | | 286143.8 |
| S.E. of regression | 116645.2 | Akaike info criterion | | 26.27262 |
| Sum squared resid | 3.40E+11 | Schwarz criterion | | 26.41535 |
| Log likelihood | -364.8167 | Hannan-Quinn criter. | | 26.31625 |
| F-statistic | 68.73976 | Durbin-Watson stat | | 2.032834 |
| Prob(F-statistic) | 0.000000 |  |  |  |
|  |  |  |  |  |
|  |  |  |  |  |
| Inverted AR Roots | .95 | -.17 | | |
|  |  |  |  |  |
|  |  |  |  |  |

| Dependent Variable: ITR | | | |  |
| --- | --- | --- | --- | --- |
| Method: Least Squares | | | |  |
| Date: 08/11/21 Time: 12:04 | | | |  |
| Sample (adjusted): 1993 2020 | | | |  |
| Included observations: 28 after adjustments | | | | |
| Convergence achieved after 8 iterations | | | | |
|  |  |  |  |  |
|  |  |  |  |  |
| Variable | Coefficient | Std. Error | t-Statistic | Prob. |
|  |  |  |  |  |
|  |  |  |  |  |
| C | 982154.5 | 263328.9 | 3.729763 | 0.0010 |
| AR(1) | 0.381929 | 0.178596 | 2.138508 | 0.0424 |
| AR(2) | 0.484308 | 0.175591 | 2.758152 | 0.0107 |
|  |  |  |  |  |
|  |  |  |  |  |
| R-squared | 0.647606 | Mean dependent var | | 774718.6 |
| Adjusted R-squared | 0.619414 | S.D. dependent var | | 149315.9 |
| S.E. of regression | 92115.41 | Akaike info criterion | | 25.80043 |
| Sum squared resid | 2.12E+11 | Schwarz criterion | | 25.94317 |
| Log likelihood | -358.2060 | Hannan-Quinn criter. | | 25.84406 |
| F-statistic | 22.97164 | Durbin-Watson stat | | 2.129365 |
| Prob(F-statistic) | 0.000002 |  |  |  |
|  |  |  |  |  |
|  |  |  |  |  |
| Inverted AR Roots | .91 | -.53 | | |
|  |  |  |  |  |
|  |  |  |  |  |

| Dependent Variable: TTR | | | |  |
| --- | --- | --- | --- | --- |
| Method: Two-Stage Least Squares | | | |  |
| Date: 08/11/21 Time: 12:12 | | | |  |
| Sample (adjusted): 1992 2020 | | | |  |
| Included observations: 29 after adjustments | | | | |
| Instrument specification: TAIWANGDP(-1) | | | | |
| Constant added to instrument list | | | | |
|  |  |  |  |  |
|  |  |  |  |  |
| Variable | Coefficient | Std. Error | t-Statistic | Prob. |
|  |  |  |  |  |
|  |  |  |  |  |
| C | 362526.8 | 90342.47 | 4.012806 | 0.0004 |
| TAIWANGDP | 0.102249 | 0.006872 | 14.87832 | 0.0000 |
|  |  |  |  |  |
|  |  |  |  |  |
| R-squared | 0.892373 | Mean dependent var | | 1644841. |
| Adjusted R-squared | 0.888387 | S.D. dependent var | | 436587.0 |
| S.E. of regression | 145857.6 | Sum squared resid | | 5.74E+11 |
| F-statistic | 221.3645 | Durbin-Watson stat | | 1.493788 |
| Prob(F-statistic) | 0.000000 | Second-Stage SSR | | 6.28E+11 |
| J-statistic | 0.000000 | Instrument rank | | 2 |
|  |  |  |  |  |
|  |  |  |  |  |

| Dependent Variable: DTR | | | |  |
| --- | --- | --- | --- | --- |
| Method: Two-Stage Least Squares | | | |  |
| Date: 08/11/21 Time: 12:15 | | | |  |
| Sample (adjusted): 1992 2020 | | | |  |
| Included observations: 29 after adjustments | | | | |
| Instrument specification: TAIWANGDP(-1) | | | | |
| Constant added to instrument list | | | | |
|  |  |  |  |  |
|  |  |  |  |  |
| Variable | Coefficient | Std. Error | t-Statistic | Prob. |
|  |  |  |  |  |
|  |  |  |  |  |
| C | 18069.49 | 58461.47 | 0.309084 | 0.7596 |
| TAIWANGDP | 0.068745 | 0.004447 | 15.45805 | 0.0000 |
|  |  |  |  |  |
|  |  |  |  |  |
| R-squared | 0.898609 | Mean dependent var | | 880200.2 |
| Adjusted R-squared | 0.894854 | S.D. dependent var | | 291078.7 |
| S.E. of regression | 94385.86 | Sum squared resid | | 2.41E+11 |
| F-statistic | 238.9514 | Durbin-Watson stat | | 1.386597 |
| Prob(F-statistic) | 0.000000 | Second-Stage SSR | | 2.44E+11 |
| J-statistic | 0.000000 | Instrument rank | | 2 |
|  |  |  |  |  |
|  |  |  |  |  |

| Dependent Variable: ITR | | | |  |
| --- | --- | --- | --- | --- |
| Method: Two-Stage Least Squares | | | |  |
| Date: 08/11/21 Time: 12:17 | | | |  |
| Sample (adjusted): 1992 2020 | | | |  |
| Included observations: 29 after adjustments | | | | |
| Instrument specification: TAIWANGDP(-1) | | | | |
| Constant added to instrument list | | | | |
|  |  |  |  |  |
|  |  |  |  |  |
| Variable | Coefficient | Std. Error | t-Statistic | Prob. |
|  |  |  |  |  |
|  |  |  |  |  |
| C | 344457.3 | 48101.62 | 7.161032 | 0.0000 |
| TAIWANGDP | 0.033505 | 0.003659 | 9.156530 | 0.0000 |
|  |  |  |  |  |
|  |  |  |  |  |
| R-squared | 0.762083 | Mean dependent var | | 764641.1 |
| Adjusted R-squared | 0.753271 | S.D. dependent var | | 156346.1 |
| S.E. of regression | 77659.92 | Sum squared resid | | 1.63E+11 |
| F-statistic | 83.84204 | Durbin-Watson stat | | 1.735247 |
| Prob(F-statistic) | 0.000000 | Second-Stage SSR | | 1.79E+11 |
| J-statistic | 7.66E-43 | Instrument rank | | 2 |
|  |  |  |  |  |
|  |  |  |  |  |
