# Supplementary material for: Threshold analysis regarding the optimal tax rate and tax evasion. Empirical evidence from Taiwan
Source: PLoS One. 2023 Mar 31;18(3):e0281101. doi: 10.1371/journal.pone.0281101 (PMC10065287; doi:10.1371/journal.pone.0281101)
Supplement: S2 File — (DOCX) [file pone.0281101.s002.docx]

1. 總稅收與GDP的拐點分析

資料來源:財政部統計年報

SUROLS法

Case 1

taxrevenue=c(1)+c(2)*gdp+c(3)*squaregdp+c(4)* triplegdp

| System: UNTITLED | | |  |  |
| --- | --- | --- | --- | --- |
| Estimation Method: Seemingly Unrelated Regression | | | | |
| Date: 04/28/21 Time: 23:58 | | | |  |
| Sample: 1991 2020 | | |  |  |
| Included observations: 30 | | | |  |
| Total system (balanced) observations 30 | | | | |
| Linear estimation after one-step weighting matrix | | | | |
|  |  |  |  |  |
|  |  |  |  |  |
|  | Coefficient | Std. Error | t-Statistic | Prob. |
|  |  |  |  |  |
|  |  |  |  |  |
| C(1) | -228482.3 | 562914.2 | -0.405892 | 0.6881 |
| C(2) | 0.307130 | 0.156693 | 1.960071 | 0.0608 |
| C(3) | -2.07E-08 | 1.35E-08 | -1.540642 | 0.1355 |
| C(4) | 6.26E-16 | 3.61E-16 | 1.734142 | 0.0947 |
|  |  |  |  |  |
|  |  |  |  |  |
| Determinant residual covariance | | 1.59E+10 |  |  |
|  |  |  |  |  |
|  |  |  |  |  |
|  |  |  |  |  |
| Equation: TAXREVENUE=C(1)+C(2)*GDP+C(3)*SQUAREGDP+C(4)* | | | | |
| TRIPLEGDP | | |  |  |
| Observations: 30 | | |  |  |
| R-squared | 0.920921 | Mean dependent var | | 1616967. |
| Adjusted R-squared | 0.911796 | S.D. dependent var | | 455350.8 |
| S.E. of regression | 135235.1 | Sum squared resid | | 4.76E+11 |
| Durbin-Watson stat | 1.917645 |  |  |  |
|  |  |  |  |  |
|  |  |  |  |  |

Case 2

taxrevenue=c(1)+c(2)*gdp+c(3)*squaregdp+c(4)* triplegdp

+c(5)*debt

| System: UNTITLED | | |  |  |
| --- | --- | --- | --- | --- |
| Estimation Method: Seemingly Unrelated Regression | | | | |
| Date: 04/28/21 Time: 23:59 | | | |  |
| Sample: 1991 2020 | | |  |  |
| Included observations: 30 | | | |  |
| Total system (unbalanced) observations 58 | | | | |
| Linear estimation after one-step weighting matrix | | | | |
|  |  |  |  |  |
|  |  |  |  |  |
|  | Coefficient | Std. Error | t-Statistic | Prob. |
|  |  |  |  |  |
|  |  |  |  |  |
| C(1) | -228483.5 | 562565.1 | -0.406146 | 0.6863 |
| C(2) | 0.307130 | 0.156596 | 1.961285 | 0.0551 |
| C(3) | -2.07E-08 | 1.34E-08 | -1.541601 | 0.1291 |
| C(4) | 6.26E-16 | 3.61E-16 | 1.735225 | 0.0885 |
| C(5) | -9.45E-18 | 1.34E-15 | -0.007039 | 0.9944 |
|  |  |  |  |  |
|  |  |  |  |  |
| Determinant residual covariance | | 9.44E-14 |  |  |
|  |  |  |  |  |
|  |  |  |  |  |
|  |  |  |  |  |
| Equation: TAXREVENUE=C(1)+C(2)*GDP+C(3)*SQUAREGDP+C(4)* | | | | |
| TRIPLEGDP | | |  |  |
| Observations: 30 | | |  |  |
| R-squared | 0.920921 | Mean dependent var | | 1616967. |
| Adjusted R-squared | 0.911796 | S.D. dependent var | | 455350.8 |
| S.E. of regression | 135235.1 | Sum squared resid | | 4.76E+11 |
| Durbin-Watson stat | 1.917647 |  |  |  |
|  |  |  |  |  |
| Equation: +C(5)*DEBT | | |  |  |
| Observations: 28 | | |  |  |
| S.E. of regression | 2.49E-12 | Sum squared resid | | 1.67E-22 |
| Durbin-Watson stat | 0.226801 |  |  |  |
|  |  |  |  |  |
|  |  |  |  |  |

Case 3

taxrevenue=c(1)+c(2)*gdp+c(3)*squaregdp+c(4)* triplegdp+c(5)*debt+c(6)*consumerpriceindex

| System: UNTITLED | | |  |  |
| --- | --- | --- | --- | --- |
| Estimation Method: Seemingly Unrelated Regression | | | | |
| Date: 04/29/21 Time: 00:01 | | | |  |
| Sample: 1992 2019 | | |  |  |
| Included observations: 28 | | | |  |
| Total system (balanced) observations 28 | | | | |
| Linear estimation after one-step weighting matrix | | | | |
|  |  |  |  |  |
|  |  |  |  |  |
|  | Coefficient | Std. Error | t-Statistic | Prob. |
|  |  |  |  |  |
|  |  |  |  |  |
| C(1) | -1151395. | 1161309. | -0.991463 | 0.3322 |
| C(2) | 0.370223 | 0.227697 | 1.625945 | 0.1182 |
| C(3) | -3.08E-08 | 1.80E-08 | -1.705238 | 0.1022 |
| C(4) | 9.59E-16 | 4.83E-16 | 1.986206 | 0.0596 |
| C(5) | 0.049076 | 0.221774 | 0.221290 | 0.8269 |
| C(6) | 1127552. | 1816774. | 0.620634 | 0.5412 |
|  |  |  |  |  |
|  |  |  |  |  |
| Determinant residual covariance | | 1.44E+10 |  |  |
|  |  |  |  |  |
|  |  |  |  |  |
|  |  |  |  |  |
| Equation: TAXREVENUE=C(1)+C(2)*GDP+C(3)*SQUAREGDP+C(4)* | | | | |
| TRIPLEGDP+C(5)*DEBT+C(6)*CONSUMERPRICEINDEX | | | | |
| Observations: 28 | | |  |  |
| R-squared | 0.914893 | Mean dependent var | | 1617919. |
| Adjusted R-squared | 0.895550 | S.D. dependent var | | 419368.2 |
| S.E. of regression | 135534.5 | Sum squared resid | | 4.04E+11 |
| Durbin-Watson stat | 2.085827 |  |  |  |
|  |  |  |  |  |
|  |  |  |  |  |

1. 直接稅收與GDP的拐點分析

SUROLS法

Case1

directtaxrevenue=c(1)+c(2)*gdp+c(3)*squaregdp+c(4)* triplegdp

| System: UNTITLED | | |  |  |
| --- | --- | --- | --- | --- |
| Estimation Method: Seemingly Unrelated Regression | | | | |
| Date: 04/29/21 Time: 00:09 | | | |  |
| Sample: 1991 2020 | | |  |  |
| Included observations: 30 | | | |  |
| Total system (balanced) observations 30 | | | | |
| Linear estimation after one-step weighting matrix | | | | |
|  |  |  |  |  |
|  |  |  |  |  |
|  | Coefficient | Std. Error | t-Statistic | Prob. |
|  |  |  |  |  |
|  |  |  |  |  |
| C(1) | 140925.6 | 372868.6 | 0.377950 | 0.7085 |
| C(2) | 0.061019 | 0.103792 | 0.587900 | 0.5617 |
| C(3) | -1.52E-09 | 8.91E-09 | -0.170063 | 0.8663 |
| C(4) | 9.13E-17 | 2.39E-16 | 0.381808 | 0.7057 |
|  |  |  |  |  |
|  |  |  |  |  |
| Determinant residual covariance | | 6.95E+09 |  |  |
|  |  |  |  |  |
|  |  |  |  |  |
|  |  |  |  |  |
| Equation: DIRECTTAXREVENUE=C(1)+C(2)*GDP+C(3)*SQUAREGDP | | | | |
| +C(4)* TRIPLEGDP | | | |  |
| Observations: 30 | | |  |  |
| R-squared | 0.920913 | Mean dependent var | | 862726.0 |
| Adjusted R-squared | 0.911788 | S.D. dependent var | | 301605.1 |
| S.E. of regression | 89578.34 | Sum squared resid | | 2.09E+11 |
| Durbin-Watson stat | 1.701181 |  |  |  |
|  |  |  |  |  |
|  |  |  |  |  |

Case 2

directtaxrevenue=c(1)+c(2)*gdp+c(3)*squaregdp+c(4)* triplegdp

+c(5)*debt

| System: UNTITLED | | |  |  |
| --- | --- | --- | --- | --- |
| Estimation Method: Seemingly Unrelated Regression | | | | |
| Date: 04/29/21 Time: 00:11 | | | |  |
| Sample: 1991 2020 | | |  |  |
| Included observations: 30 | | | |  |
| Total system (unbalanced) observations 58 | | | | |
| Linear estimation after one-step weighting matrix | | | | |
|  |  |  |  |  |
|  |  |  |  |  |
|  | Coefficient | Std. Error | t-Statistic | Prob. |
|  |  |  |  |  |
|  |  |  |  |  |
| C(1) | 140925.6 | 372864.0 | 0.377954 | 0.7070 |
| C(2) | 0.061019 | 0.103791 | 0.587907 | 0.5591 |
| C(3) | -1.52E-09 | 8.91E-09 | -0.170065 | 0.8656 |
| C(4) | 9.13E-17 | 2.39E-16 | 0.381813 | 0.7041 |
| C(5) | -3.62E-22 | 2.62E-18 | -0.000138 | 0.9999 |
|  |  |  |  |  |
|  |  |  |  |  |
| Determinant residual covariance | | 6.09E-23 |  |  |
|  |  |  |  |  |
|  |  |  |  |  |
|  |  |  |  |  |
| Equation: DIRECTTAXREVENUE=C(1)+C(2)*GDP+C(3)*SQUAREGDP | | | | |
| +C(4)* TRIPLEGDP | | | |  |
| Observations: 30 | | |  |  |
| R-squared | 0.920913 | Mean dependent var | | 862726.0 |
| Adjusted R-squared | 0.911788 | S.D. dependent var | | 301605.1 |
| S.E. of regression | 89578.34 | Sum squared resid | | 2.09E+11 |
| Durbin-Watson stat | 1.701181 |  |  |  |
|  |  |  |  |  |
| Equation: +C(5)*DEBT | | |  |  |
| Observations: 28 | | |  |  |
| S.E. of regression | 9.53E-17 | Sum squared resid | | 2.45E-31 |
| Durbin-Watson stat | 0.226801 |  |  |  |
|  |  |  |  |  |
|  |  |  |  |  |

Case 3
directtaxrevenue=c(1)+c(2)*gdp+c(3)*squaregdp+c(4)* triplegdp

+c(5)*debt+c(6)*consumerpriceindex

| System: UNTITLED | | |  |  |
| --- | --- | --- | --- | --- |
| Estimation Method: Seemingly Unrelated Regression | | | | |
| Date: 04/29/21 Time: 00:12 | | | |  |
| Sample: 1991 2020 | | |  |  |
| Included observations: 30 | | | |  |
| Total system (unbalanced) observations 58 | | | | |
| Linear estimation after one-step weighting matrix | | | | |
|  |  |  |  |  |
|  |  |  |  |  |
|  | Coefficient | Std. Error | t-Statistic | Prob. |
|  |  |  |  |  |
|  |  |  |  |  |
| C(1) | 140906.0 | 368020.2 | 0.382876 | 0.7034 |
| C(2) | 0.061041 | 0.102443 | 0.595857 | 0.5539 |
| C(3) | -1.52E-09 | 8.80E-09 | -0.172647 | 0.8636 |
| C(4) | 9.13E-17 | 2.36E-16 | 0.386979 | 0.7004 |
| C(5) | -2.68E-16 | 1.93E-15 | -0.138657 | 0.8903 |
| C(6) | 8.91E-11 | 5.56E-10 | 0.160154 | 0.8734 |
|  |  |  |  |  |
|  |  |  |  |  |
| Determinant residual covariance | | 9.65E-12 |  |  |
|  |  |  |  |  |
|  |  |  |  |  |
|  |  |  |  |  |
| Equation: DIRECTTAXREVENUE=C(1)+C(2)*GDP+C(3)*SQUAREGDP | | | | |
| +C(4)* TRIPLEGDP | | | |  |
| Observations: 30 | | |  |  |
| R-squared | 0.920913 | Mean dependent var | | 862726.0 |
| Adjusted R-squared | 0.911787 | S.D. dependent var | | 301605.1 |
| S.E. of regression | 89578.69 | Sum squared resid | | 2.09E+11 |
| Durbin-Watson stat | 1.701136 |  |  |  |
|  |  |  |  |  |
| Equation: +C(5)*DEBT+C(6)*CONSUMERPRICEINDEX | | | | |
| Observations: 28 | | |  |  |
| S.E. of regression | 3.92E-11 | Sum squared resid | | 4.01E-20 |
| Durbin-Watson stat | 0.767300 |  |  |  |
|  |  |  |  |  |
|  |  |  |  |  |

3.間接稅收與GDP的拐點分析

SUROLS法

Case 1

nondirecttaxrevenue=c(1)+c(2)*gdp+c(3)*squaregdp+c(4)* triplegdp

| System: UNTITLED | | |  |  |
| --- | --- | --- | --- | --- |
| Estimation Method: Seemingly Unrelated Regression | | | | |
| Date: 04/29/21 Time: 00:13 | | | |  |
| Sample: 1991 2020 | | |  |  |
| Included observations: 30 | | | |  |
| Total system (balanced) observations 30 | | | | |
| Linear estimation after one-step weighting matrix | | | | |
|  |  |  |  |  |
|  |  |  |  |  |
|  | Coefficient | Std. Error | t-Statistic | Prob. |
|  |  |  |  |  |
|  |  |  |  |  |
| C(1) | -369410.2 | 291313.4 | -1.268085 | 0.2160 |
| C(2) | 0.246111 | 0.081090 | 3.035027 | 0.0054 |
| C(3) | -1.92E-08 | 6.96E-09 | -2.759365 | 0.0105 |
| C(4) | 5.34E-16 | 1.87E-16 | 2.862245 | 0.0082 |
|  |  |  |  |  |
|  |  |  |  |  |
| Determinant residual covariance | | 4.24E+09 |  |  |
|  |  |  |  |  |
|  |  |  |  |  |
|  |  |  |  |  |
| Equation: NONDIRECTTAXREVENUE=C(1)+C(2)*GDP+C(3) | | | | |
| *SQUAREGDP+C(4)* TRIPLEGDP | | | | |
| Observations: 30 | | |  |  |
| R-squared | 0.836427 | Mean dependent var | | 754240.8 |
| Adjusted R-squared | 0.817553 | S.D. dependent var | | 163847.2 |
| S.E. of regression | 69985.45 | Sum squared resid | | 1.27E+11 |
| Durbin-Watson stat | 2.257293 |  |  |  |
|  |  |  |  |  |
|  |  |  |  |  |

case 2

nondirecttaxrevenue=c(1)+c(2)*gdp+c(3)*squaregdp+c(4)* triplegdp+c(5)*debt

| System: UNTITLED | | |  |  |
| --- | --- | --- | --- | --- |
| Estimation Method: Seemingly Unrelated Regression | | | | |
| Date: 04/29/21 Time: 00:14 | | | |  |
| Sample: 1992 2019 | | |  |  |
| Included observations: 28 | | | |  |
| Total system (balanced) observations 28 | | | | |
| Linear estimation after one-step weighting matrix | | | | |
|  |  |  |  |  |
|  |  |  |  |  |
|  | Coefficient | Std. Error | t-Statistic | Prob. |
|  |  |  |  |  |
|  |  |  |  |  |
| C(1) | -634568.6 | 414802.7 | -1.529808 | 0.1397 |
| C(2) | 0.316575 | 0.111854 | 2.830257 | 0.0095 |
| C(3) | -2.57E-08 | 9.58E-09 | -2.685242 | 0.0132 |
| C(4) | 7.23E-16 | 2.59E-16 | 2.793251 | 0.0103 |
| C(5) | 0.110728 | 0.120370 | 0.919894 | 0.3672 |
|  |  |  |  |  |
|  |  |  |  |  |
| Determinant residual covariance | | 4.28E+09 |  |  |
|  |  |  |  |  |
|  |  |  |  |  |
|  |  |  |  |  |
| Equation: NONDIRECTTAXREVENUE=C(1)+C(2)*GDP+C(3) | | | | |
| *SQUAREGDP+C(4)* TRIPLEGDP+C(5)*DEBT | | | | |
| Observations: 28 | | |  |  |
| R-squared | 0.795167 | Mean dependent var | | 753575.7 |
| Adjusted R-squared | 0.759544 | S.D. dependent var | | 147198.6 |
| S.E. of regression | 72180.73 | Sum squared resid | | 1.20E+11 |
| Durbin-Watson stat | 2.241827 |  |  |  |
|  |  |  |  |  |
|  |  |  |  |  |

Case 3
nondirecttaxrevenue=c(1)+c(2)*gdp+c(3)*squaregdp+c(4)* triplegdp +c(5)*debt+c(6)*consumerpriceindex

| System: UNTITLED | | |  |  |
| --- | --- | --- | --- | --- |
| Estimation Method: Seemingly Unrelated Regression | | | | |
| Date: 04/29/21 Time: 00:15 | | | |  |
| Sample: 1992 2019 | | |  |  |
| Included observations: 28 | | | |  |
| Total system (balanced) observations 28 | | | | |
| Linear estimation after one-step weighting matrix | | | | |
|  |  |  |  |  |
|  |  |  |  |  |
|  | Coefficient | Std. Error | t-Statistic | Prob. |
|  |  |  |  |  |
|  |  |  |  |  |
| C(1) | -650136.2 | 632358.9 | -1.028113 | 0.3151 |
| C(2) | 0.314830 | 0.123986 | 2.539233 | 0.0187 |
| C(3) | -2.56E-08 | 9.82E-09 | -2.610568 | 0.0160 |
| C(4) | 7.22E-16 | 2.63E-16 | 2.744469 | 0.0118 |
| C(5) | 0.110410 | 0.120761 | 0.914291 | 0.3705 |
| C(6) | 32265.64 | 989274.4 | 0.032615 | 0.9743 |
|  |  |  |  |  |
|  |  |  |  |  |
| Determinant residual covariance | | 4.28E+09 |  |  |
|  |  |  |  |  |
|  |  |  |  |  |
|  |  |  |  |  |
| Equation: NONDIRECTTAXREVENUE=C(1)+C(2)*GDP+C(3) | | | | |
| *SQUAREGDP+C(4)* TRIPLEGDP +C(5)*DEBT+C(6) | | | | |
| *CONSUMERPRICEINDEX | | | |  |
| Observations: 28 | | |  |  |
| R-squared | 0.795175 | Mean dependent var | | 753575.7 |
| Adjusted R-squared | 0.748624 | S.D. dependent var | | 147198.6 |
| S.E. of regression | 73801.57 | Sum squared resid | | 1.20E+11 |
| Durbin-Watson stat | 2.245922 |  |  |  |
|  |  |  |  |  |
|  |  |  |  |  |

以上case發現GDP-Taxrevenue 全部都是N型關係
　 所得稅小計 遺產及贈與稅 土地稅小計 房屋稅 契稅 直接稅合計

80年度 193,742,764 6,495,694 121,623,944 26,043,856 8,066,914 355,973,172

81年度 216,150,460 12,874,176 217,645,115 28,300,746 10,182,057 485,152,554

82年度 237,618,241 13,177,623 222,039,199 29,243,895 10,949,349 513,028,307

83年度 266,020,141 19,292,293 206,379,137 32,702,952 14,151,581 538,546,104

84年度 319,383,860 21,457,740 193,615,439 35,871,143 17,615,701 587,943,883

85年度 343,550,020 24,453,008 158,134,395 38,850,039 17,153,317 582,140,779

86年度 352,890,477 24,326,360 174,782,988 41,594,479 15,506,076 609,100,380

87年度 398,390,232 25,519,415 173,802,985 43,800,029 14,347,022 655,859,683

88年度 432,388,876 21,568,379 148,313,404 45,661,086 13,096,702 661,028,447

89年度 610,580,021 39,135,081 215,532,765 48,435,990 15,267,197 928,951,054

90年度 478,636,373 22,710,416 93,182,039 48,055,633 8,492,646 651,077,107

91年度 392,939,078 23,537,164 98,069,452 46,463,784 10,261,814 562,031,292

92年度 411,086,972 30,106,227 111,802,984 48,012,750 11,600,349 612,609,282

93年度 455,913,822 29,047,708 133,893,333 49,323,149 12,936,962 681,114,974

94年度 625,807,052 30,450,904 135,370,017 50,876,888 13,178,008 882,682,869

95年度 646,217,799 28,693,738 131,207,704 52,493,513 14,013,856 872,626,610

96年度 730,159,866 28,481,129 133,691,191 53,882,845 13,636,896 959,851,927

97年度 834,988,400 28,977,696 116,082,367 55,343,250 12,696,095 1,048,087,808

98年度 640,966,779 22,327,164 112,366,946 56,346,975 13,065,855 845,073,719

99年度 590,387,476 40,329,876 136,356,984 58,202,695 13,815,457 839,092,488

100年度 710,191,056 23,658,842 141,981,922 59,466,872 12,640,465 947,939,157

101年度 760,809,633 28,280,408 143,848,851 61,796,279 11,693,005 1,006,428,176

102年度 743,289,569 23,727,917 174,080,706 63,013,454 13,560,334 1,017,671,980

103年度 813,483,965 25,444,324 173,174,512 64,672,878 12,534,737 1,089,310,416

104年度 936,730,879 32,735,575 184,598,397 69,421,961 13,826,660 1,237,313,472

105年度 1,006,360,404 47,515,086 177,273,904 72,962,850 11,375,814 1,315,488,058

106年度 986,412,046 51,085,366 188,638,208 76,723,547 12,789,424 1,215,648,591

107年度 1,077,079,297 31,824,830 182,436,812 78,588,425 13,365,520 1,383,294,884

108年度 1,148,814,059 34,925,726 193,033,959 80,972,489 14,773,001 1,472,519,234

109年度 981,707,165 42,296,348 204,742,200 79,315,449 16,147,441 1,324,208,603

1.總稅收門檻模型

taxrevenue c gdp squaregdp

門檻變數 taxburdenratio


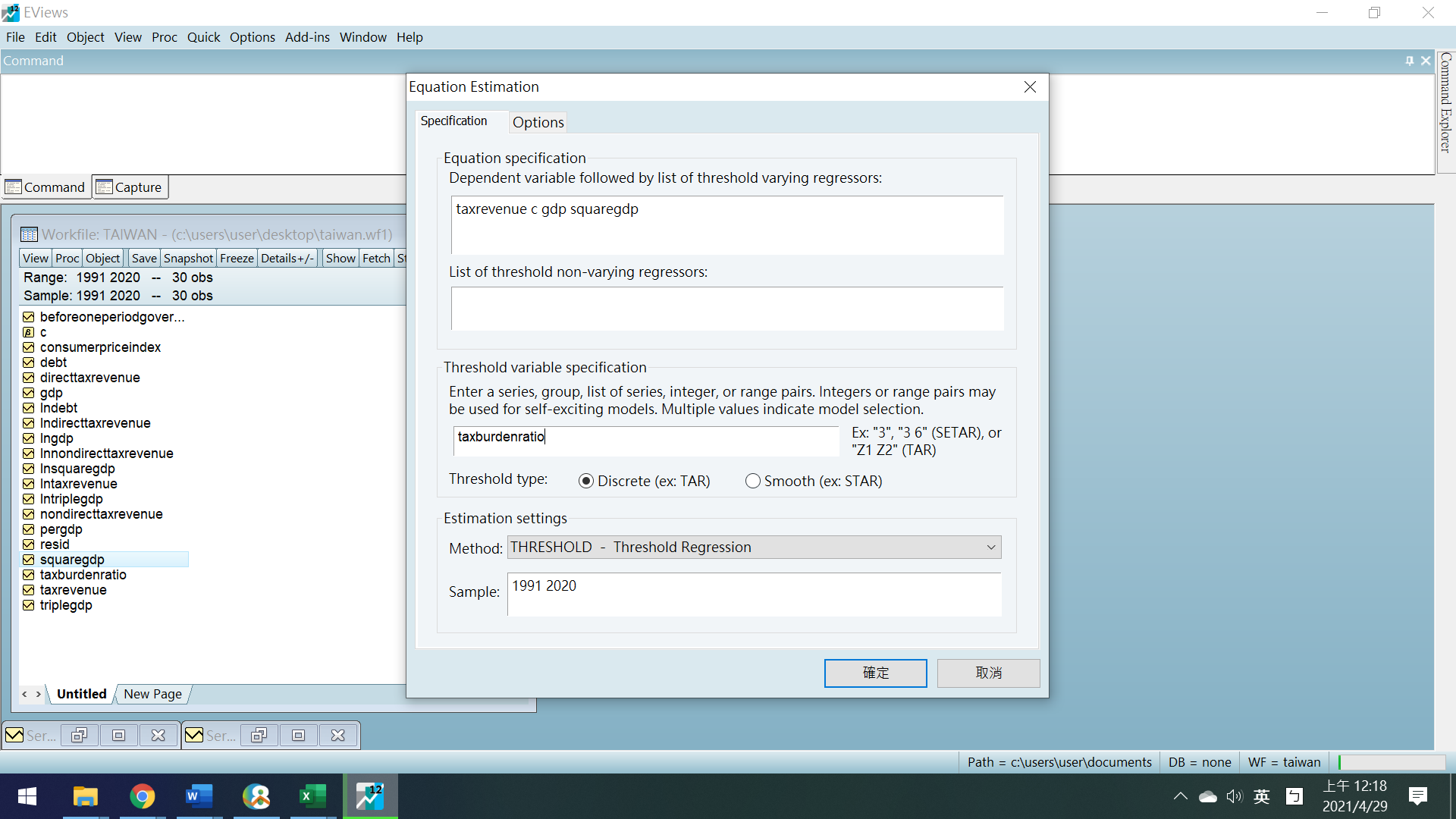


| Dependent Variable: TAXREVENUE | | | |  |
| --- | --- | --- | --- | --- |
| Method: Discrete Threshold Regression | | | | |
| Date: 04/29/21 Time: 00:19 | | | |  |
| Sample: 1991 2020 | | |  |  |
| Included observations: 30 | | | |  |
| Selection: Trimming 0.15, Max. thresholds 5, Sig. level 0.05 | | | | |
| Threshold variable: TAXBURDENRATIO | | | | |
|  |  |  |  |  |
|  |  |  |  |  |
| Variable | Coefficient | Std. Error | t-Statistic | Prob. |
|  |  |  |  |  |
|  |  |  |  |  |
| TAXBURDENRATIO < 12.5 -- 11 obs | | | | |
|  |  |  |  |  |
|  |  |  |  |  |
| C | -53945.39 | 372251.1 | -0.144917 | 0.8862 |
| GDP | 0.122341 | 0.052241 | 2.341852 | 0.0291 |
| SQUAREGDP | 1.07E-10 | 1.78E-09 | 0.059753 | 0.9529 |
|  |  |  |  |  |
|  |  |  |  |  |
| 12.5 <= TAXBURDENRATIO < 13 -- 5 obs | | | | |
|  |  |  |  |  |
|  |  |  |  |  |
| C | 7330704. | 1027003. | 7.137959 | 0.0000 |
| GDP | -0.856864 | 0.150661 | -5.687351 | 0.0000 |
| SQUAREGDP | 3.22E-08 | 5.27E-09 | 6.099663 | 0.0000 |
|  |  |  |  |  |
|  |  |  |  |  |
| 13 <= TAXBURDENRATIO -- 14 obs | | | | |
|  |  |  |  |  |
|  |  |  |  |  |
| C | 475913.8 | 105316.4 | 4.518894 | 0.0002 |
| GDP | 0.080666 | 0.019707 | 4.093196 | 0.0005 |
| SQUAREGDP | 1.25E-09 | 8.12E-10 | 1.543364 | 0.1377 |
|  |  |  |  |  |
|  |  |  |  |  |
| R-squared | 0.990176 | Mean dependent var | | 1616967. |
| Adjusted R-squared | 0.986433 | S.D. dependent var | | 455350.8 |
| S.E. of regression | 53037.55 | Akaike info criterion | | 24.83871 |
| Sum squared resid | 5.91E+10 | Schwarz criterion | | 25.25907 |
| Log likelihood | -363.5807 | Hannan-Quinn criter. | | 24.97319 |
| F-statistic | 264.5731 | Durbin-Watson stat | | 1.658428 |
| Prob(F-statistic) | 0.000000 |  |  |  |
|  |  |  |  |  |
|  |  |  |  |  |

(1)12.5> TAXBURDENRATIO時,所得變動對稅收具正向影響。

(2)13>TAXBURDENRATIO > 12.5時, 所得增加導致稅收不增反降，有稅基侵蝕。

(3) 13 < TAXBURDENRATIO時,所得變動對稅收具正向影響。

因為在第三階段的

直接稅(negative effect)+與間接稅(positive effect)，間接稅的效果大於直接稅，成就了GDP上升, 總稅收增加的結果。


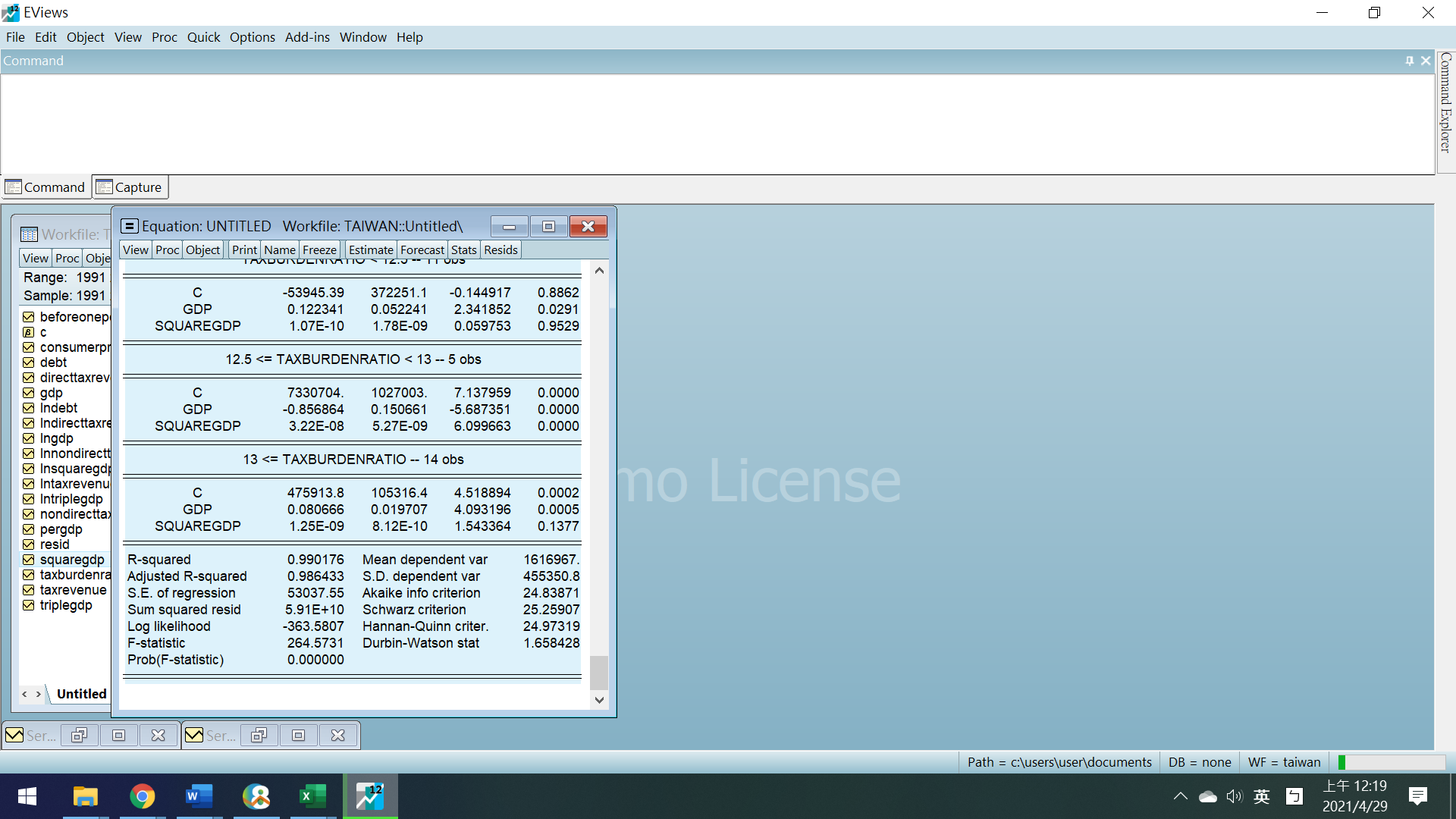


2.直接稅門檻模型

directtaxrevenue c gdp squaregdp

門檻變數 taxburdenratio

| Dependent Variable: DIRECTTAXREVENUE | | | | |
| --- | --- | --- | --- | --- |
| Method: Discrete Threshold Regression | | | | |
| Date: 04/29/21 Time: 00:20 | | | |  |
| Sample: 1991 2020 | | |  |  |
| Included observations: 30 | | | |  |
| Selection: Trimming 0.15, Max. thresholds 5, Sig. level 0.05 | | | | |
| Threshold variable: TAXBURDENRATIO | | | | |
|  |  |  |  |  |
|  |  |  |  |  |
| Variable | Coefficient | Std. Error | t-Statistic | Prob. |
|  |  |  |  |  |
|  |  |  |  |  |
| TAXBURDENRATIO < 12.599999 -- 13 obs | | | | |
|  |  |  |  |  |
|  |  |  |  |  |
| C | -572825.9 | 322047.7 | -1.778699 | 0.0898 |
| GDP | 0.128270 | 0.045222 | 2.836426 | 0.0099 |
| SQUAREGDP | -1.57E-09 | 1.54E-09 | -1.017246 | 0.3206 |
|  |  |  |  |  |
|  |  |  |  |  |
| 12.599999 <= TAXBURDENRATIO < 13.399999 -- 7 obs | | | | |
|  |  |  |  |  |
|  |  |  |  |  |
| C | 2306035. | 623148.3 | 3.700620 | 0.0013 |
| GDP | -0.244948 | 0.087008 | -2.815226 | 0.0104 |
| SQUAREGDP | 1.06E-08 | 2.91E-09 | 3.655764 | 0.0015 |
|  |  |  |  |  |
|  |  |  |  |  |
| 13.399999 <= TAXBURDENRATIO -- 10 obs | | | | |
|  |  |  |  |  |
|  |  |  |  |  |
| C | 369262.3 | 181478.2 | 2.034747 | 0.0547 |
| GDP | -0.011211 | 0.042543 | -0.263531 | 0.7947 |
| SQUAREGDP | 4.69E-09 | 2.36E-09 | 1.987062 | 0.0601 |
|  |  |  |  |  |
|  |  |  |  |  |
| R-squared | 0.982957 | Mean dependent var | | 862726.0 |
| Adjusted R-squared | 0.976464 | S.D. dependent var | | 301605.1 |
| S.E. of regression | 46270.56 | Akaike info criterion | | 24.56572 |
| Sum squared resid | 4.50E+10 | Schwarz criterion | | 24.98608 |
| Log likelihood | -359.4859 | Hannan-Quinn criter. | | 24.70020 |
| F-statistic | 151.3946 | Durbin-Watson stat | | 1.805336 |
| Prob(F-statistic) | 0.000000 |  |  |  |
|  |  |  |  |  |
|  |  |  |  |  |


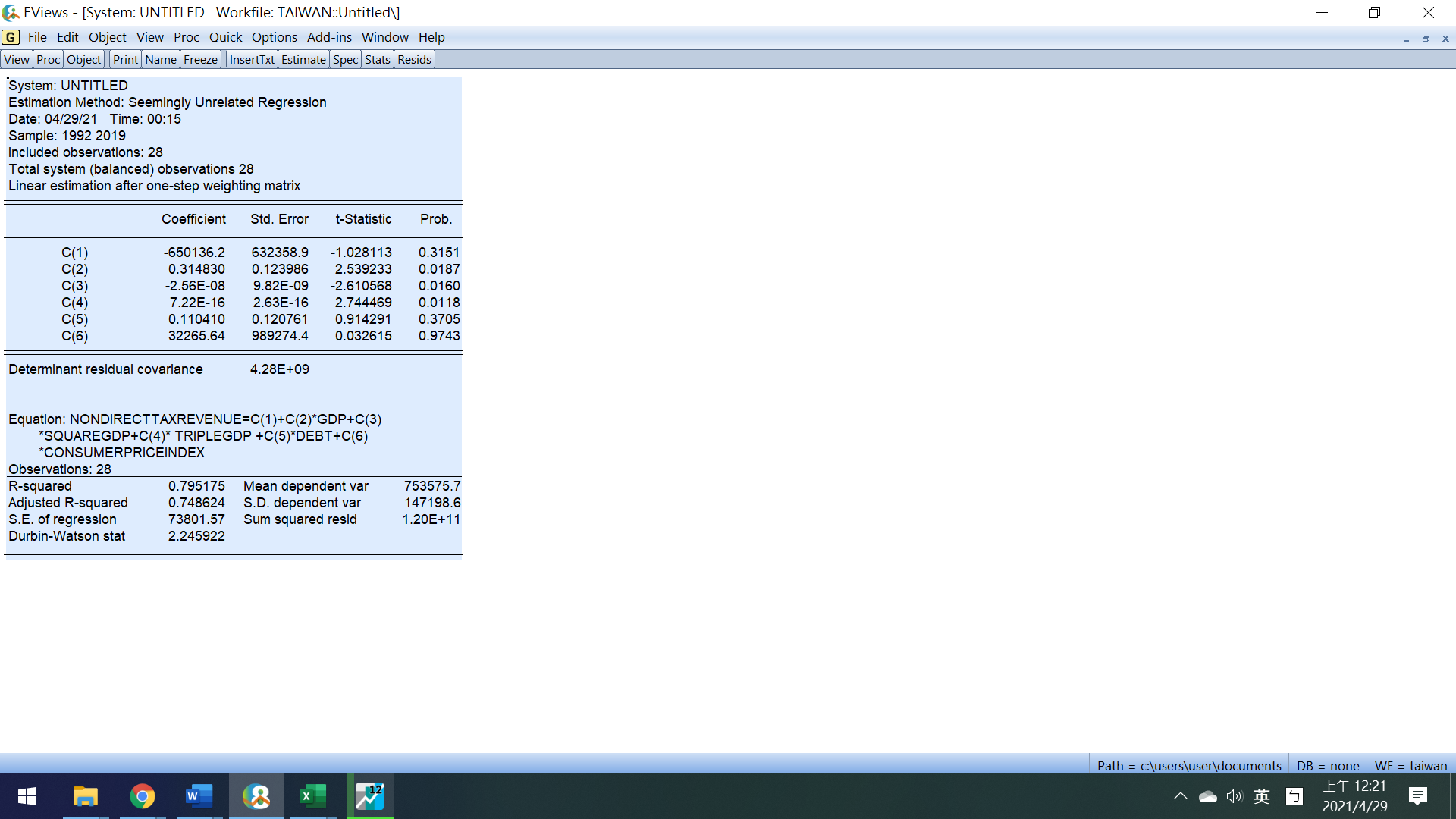


表示

(1)12.599999 <= TAXBURDENRATIO時,所得變動對直接稅具正向影響。

(2)12.599999 <= TAXBURDENRATIO < 13.399999 -- 7 obs時, 所得增加導致直接稅收不增反降，有稅基侵蝕。

(3)TAXBURDENRATIO > 13.39時, 所得增加導致直接稅收不增反降，有稅基侵蝕。

3.間接稅門檻模型

nondirecttaxrevenue c gdp squaregdp

門檻變數 taxburdenratio


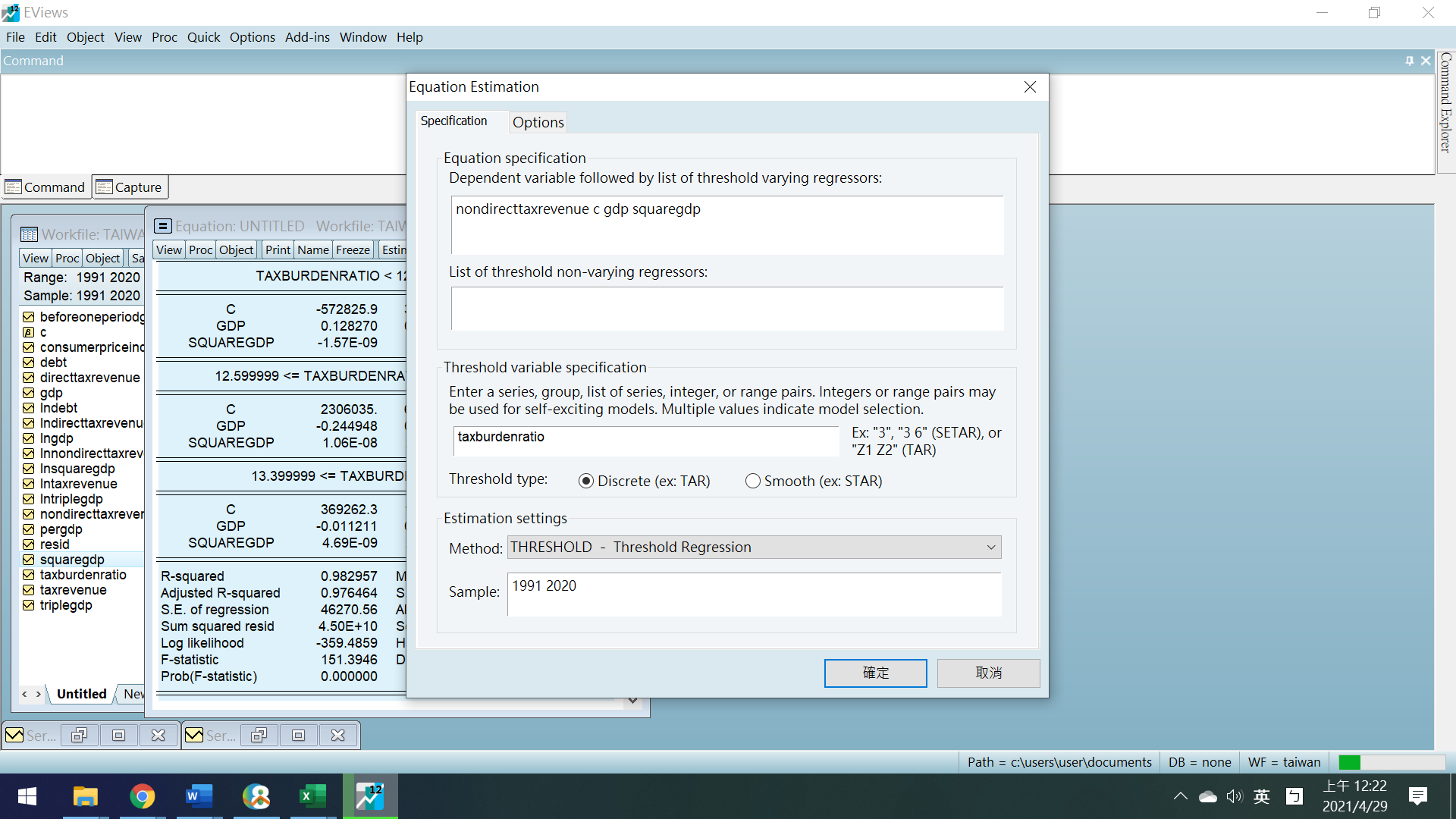


| Dependent Variable: NONDIRECTTAXREVENUE | | | | |
| --- | --- | --- | --- | --- |
| Method: Discrete Threshold Regression | | | | |
| Date: 04/29/21 Time: 00:22 | | | |  |
| Sample: 1991 2020 | | |  |  |
| Included observations: 30 | | | |  |
| No thresholds selected | | | |  |
| Selection: Trimming 0.15, Max. thresholds 5, Sig. level 0.05 | | | | |
|  |  |  |  |  |
|  |  |  |  |  |
| Variable | Coefficient | Std. Error | t-Statistic | Prob. |
|  |  |  |  |  |
|  |  |  |  |  |
| C | 411809.9 | 121099.8 | 3.400582 | 0.0021 |
| GDP | 0.019420 | 0.020700 | 0.938170 | 0.3565 |
| SQUAREGDP | 6.18E-10 | 8.23E-10 | 0.750320 | 0.4596 |
|  |  |  |  |  |
|  |  |  |  |  |
| R-squared | 0.791758 | Mean dependent var | | 754240.8 |
| Adjusted R-squared | 0.776332 | S.D. dependent var | | 163847.2 |
| S.E. of regression | 77489.10 | Akaike info criterion | | 25.44830 |
| Sum squared resid | 1.62E+11 | Schwarz criterion | | 25.58842 |
| Log likelihood | -378.7245 | Hannan-Quinn criter. | | 25.49313 |
| F-statistic | 51.32832 | Durbin-Watson stat | | 1.744366 |
| Prob(F-statistic) | 0.000000 |  |  |  |
|  |  |  |  |  |
|  |  |  |  |  |

以上分析顯示間接稅並沒有門檻效果,也就是隨著GDP的增加, 間接稅也增加, GDP與間接稅兩者呈現倒U型關係


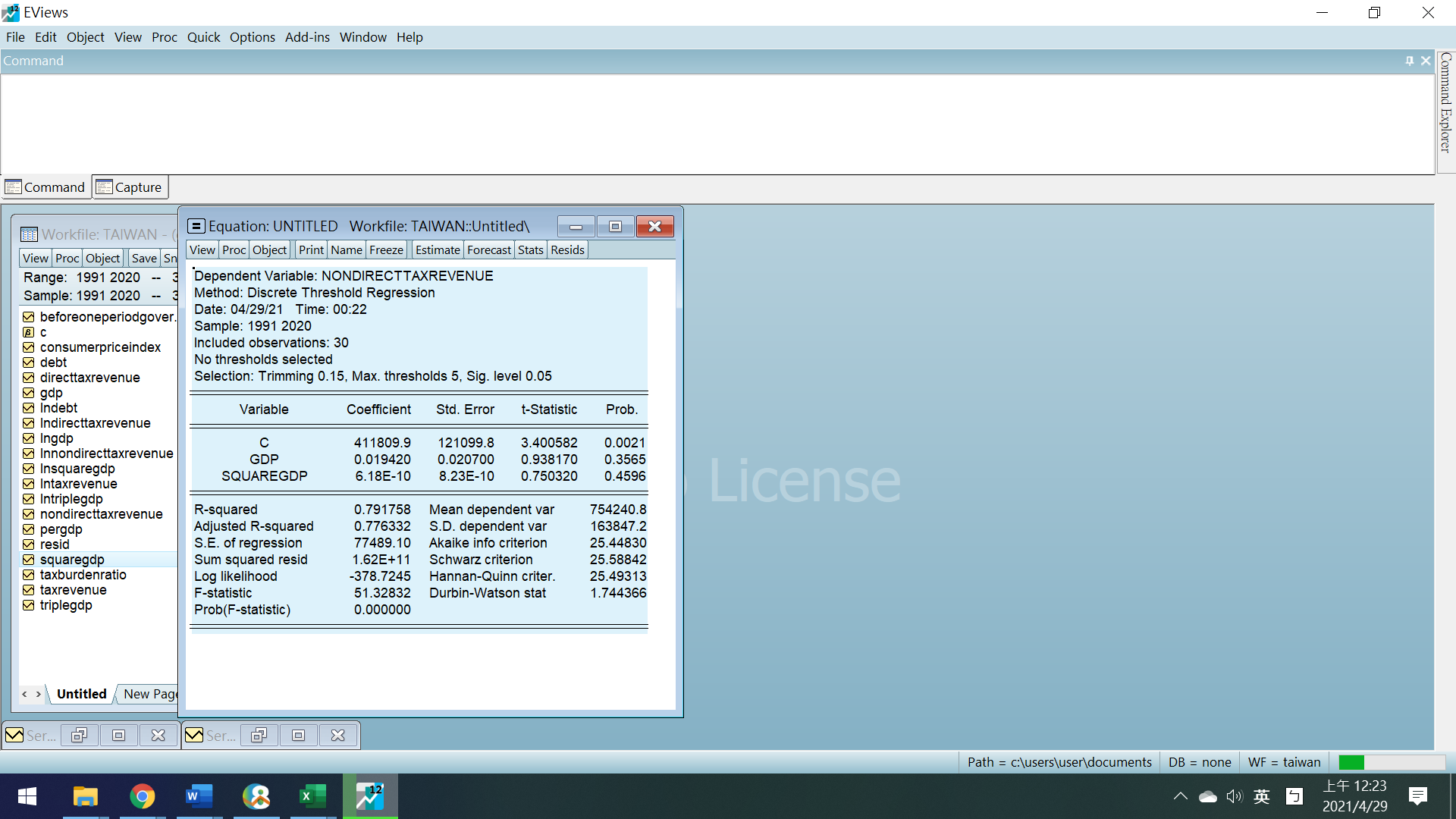


以下是用Mathlab求多項式的臨界點(兩根)

可與2020年台灣GDP多少作比較 觀察是否已經到達拐點 若否表示稅收與GDP呈正相關

(1)總稅收與GDP的Matlab分析

可知X 對y1= (6.26*exp(-16)*x^3-(2.07*exp(-0.8)*x^2+0.30713*x-228482.3 作一階導數

得

3* (6.26*exp(-16)*x^2-2*(2.07*exp(-0.8)*x+0.30713=0

在Mathlab輸入資料如下 可得臨界點x1,x2如下

X1=8.8020e+05

X2=0.1651


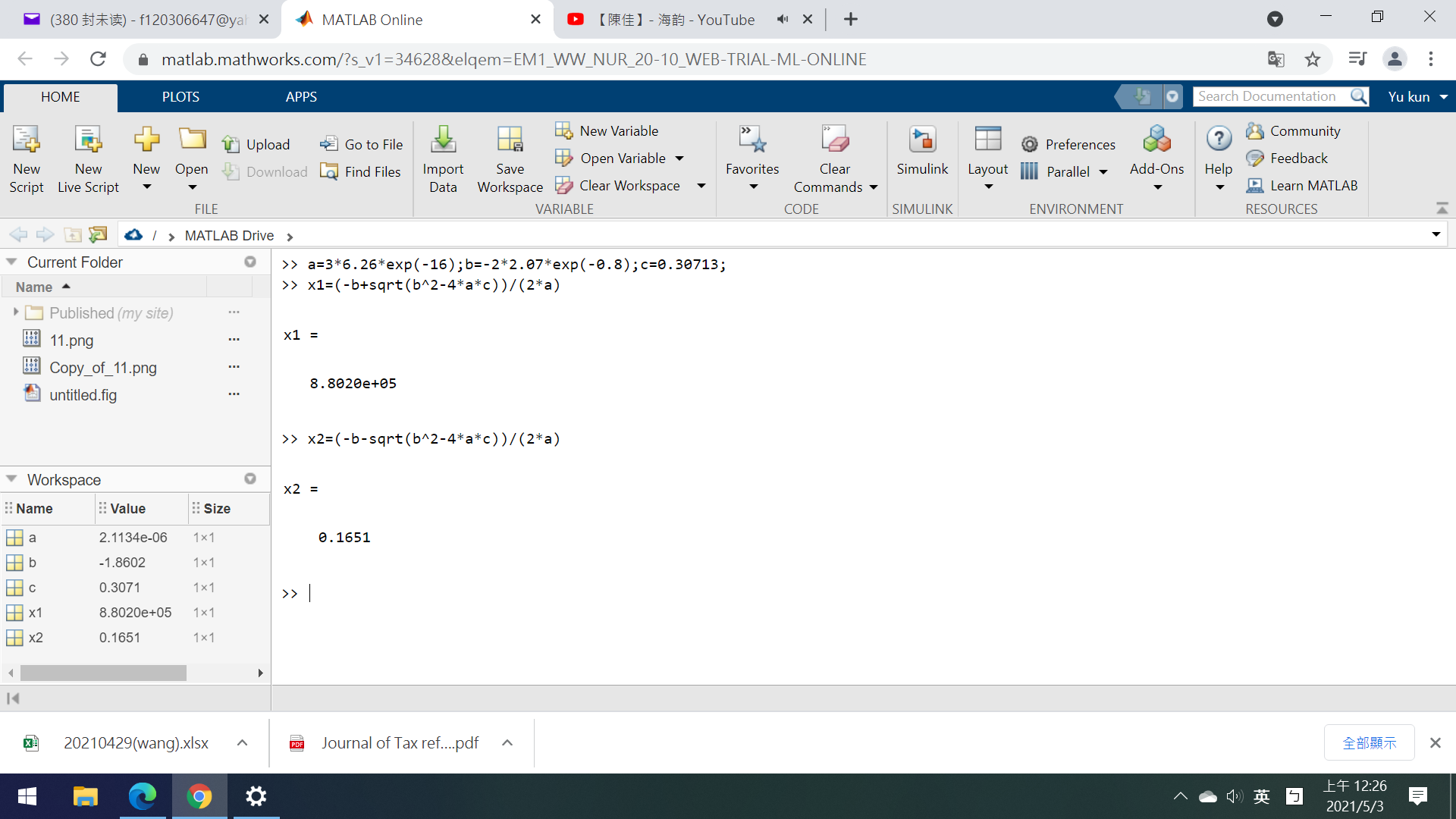


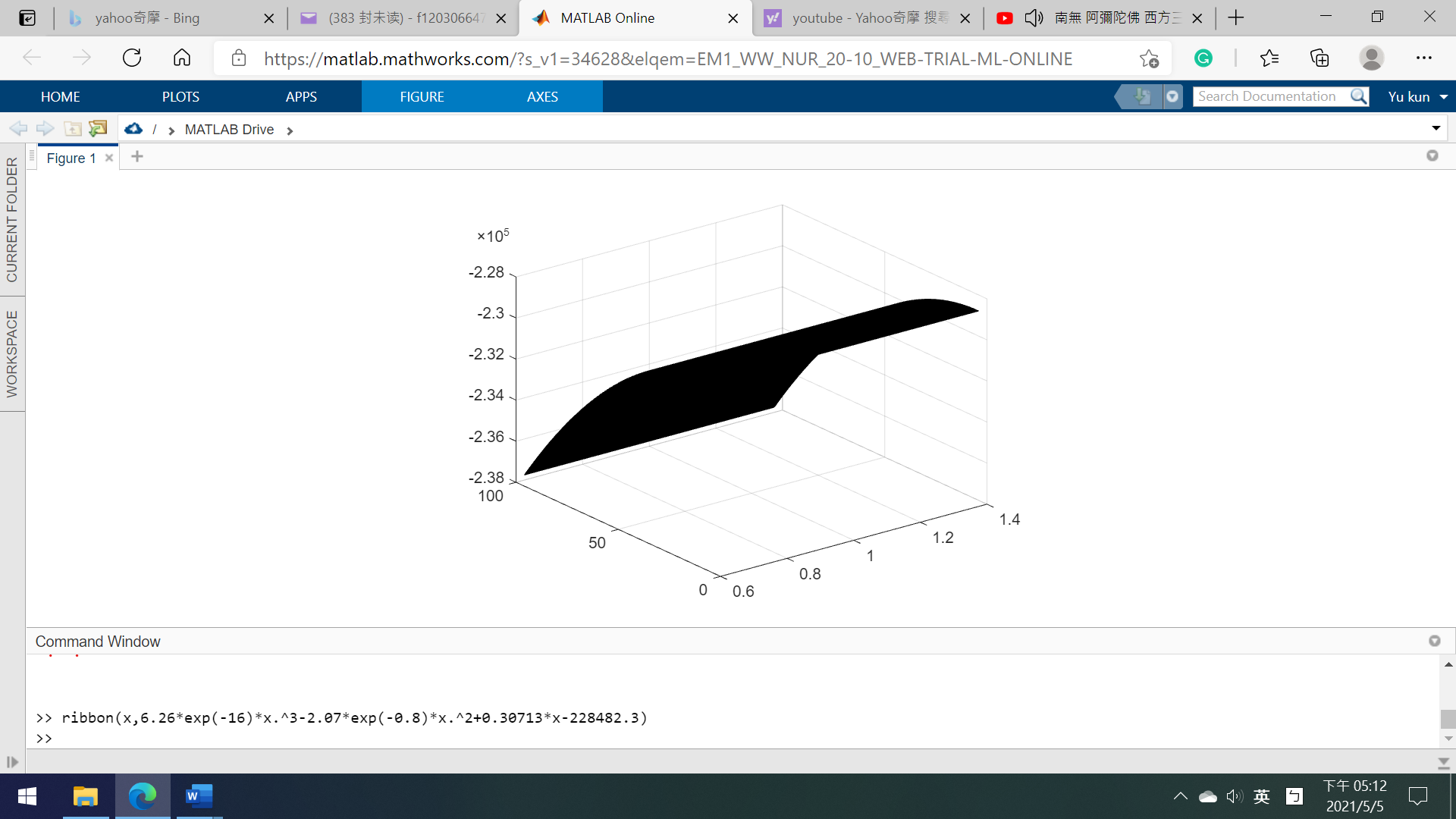


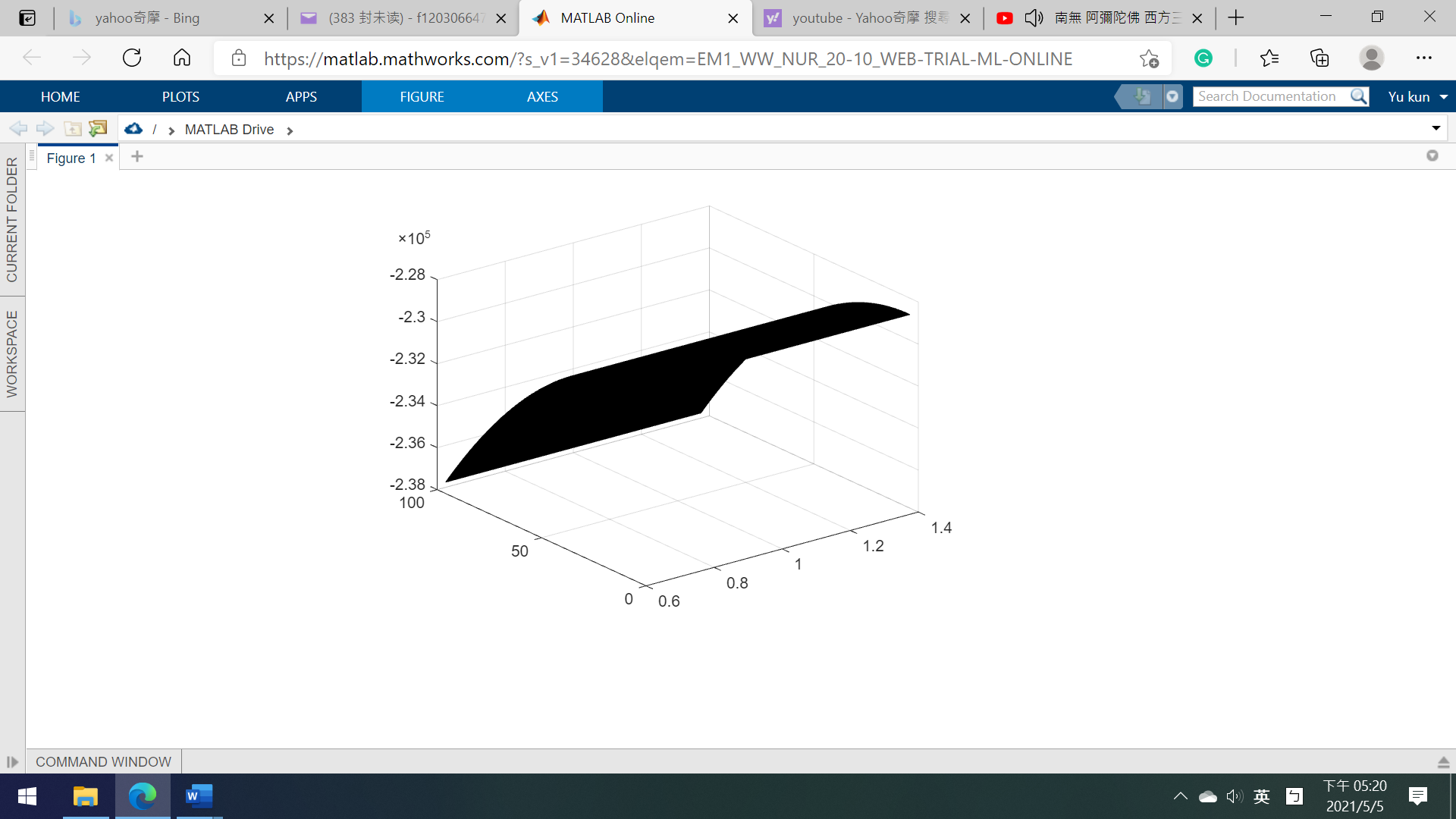


圖一:TTR-GDP,Taiwan,1990-2020

(2)直接稅與GDP的Matlab分析

可知X 對y2= (9.13*exp(-17)*x^3-(1.52*exp(-0.9)*x^2+0.061019*x+140925.6 作一階導數

得

3* (9.13*exp(-17)*x^2-2*(1.52*exp(-0.9)*x+0.061019=0

在Mathlab輸入資料如下 可得臨界點x1,x2如下

X1=-0.0494

X2=-1.0900e+06


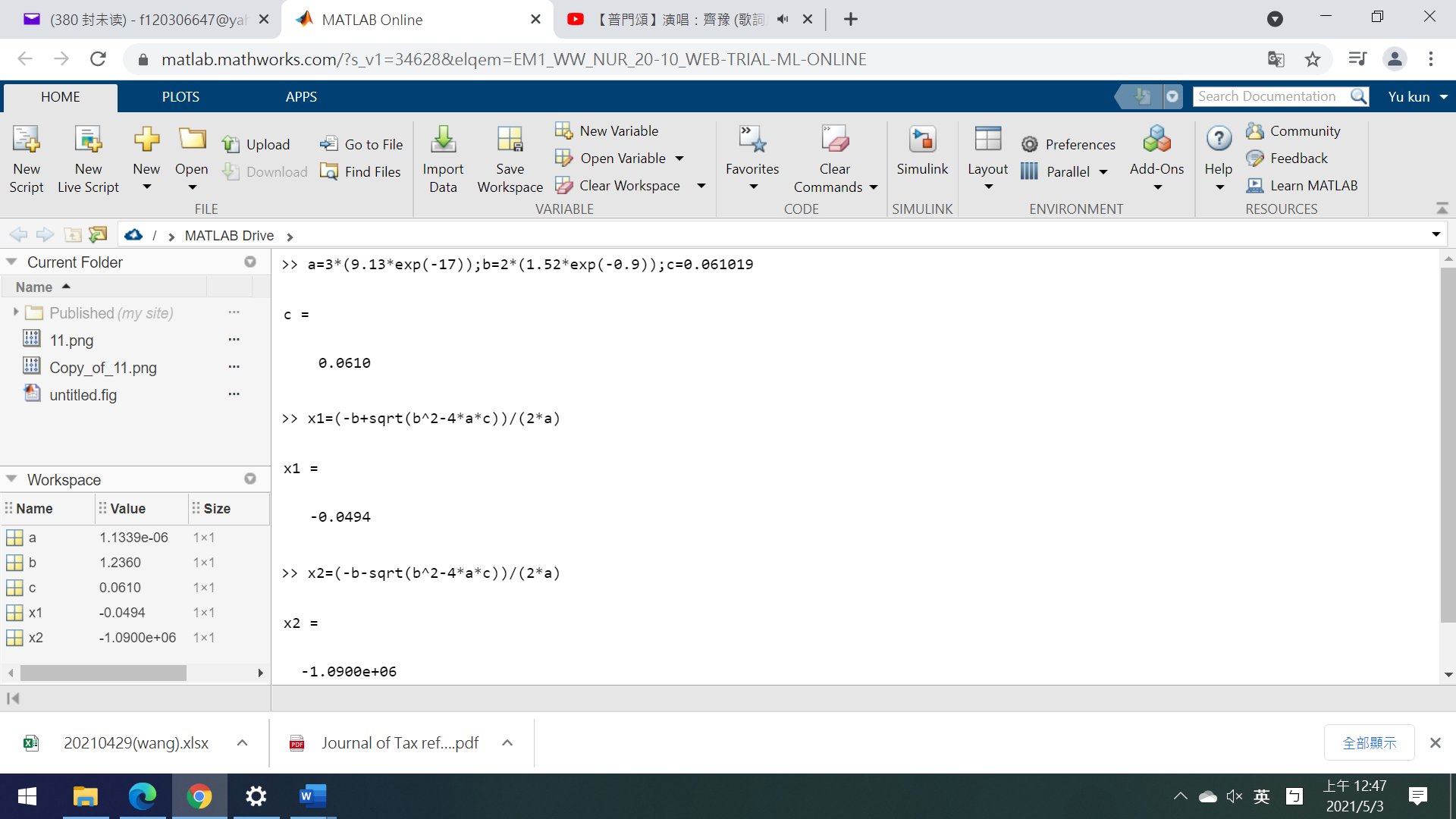


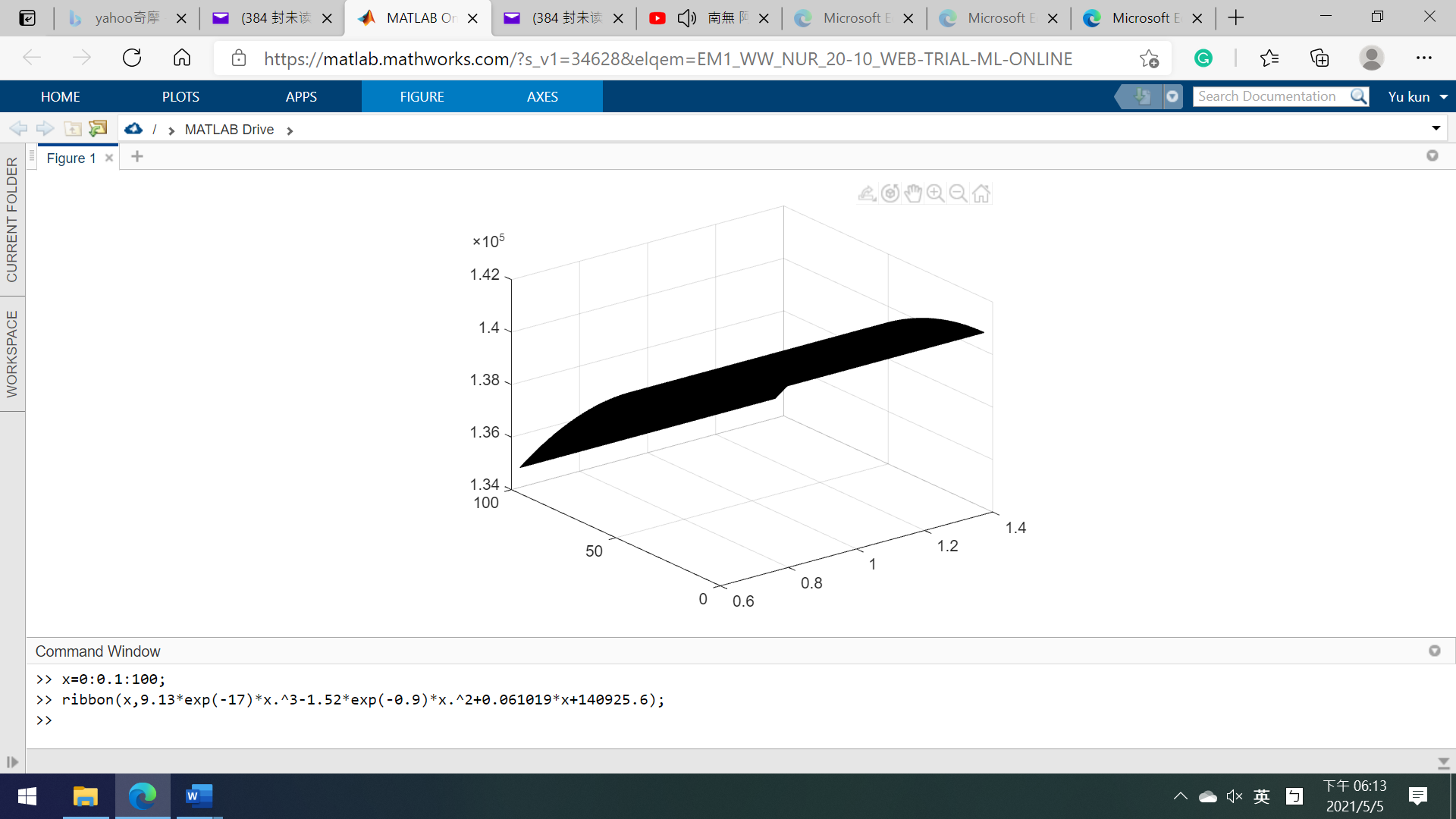


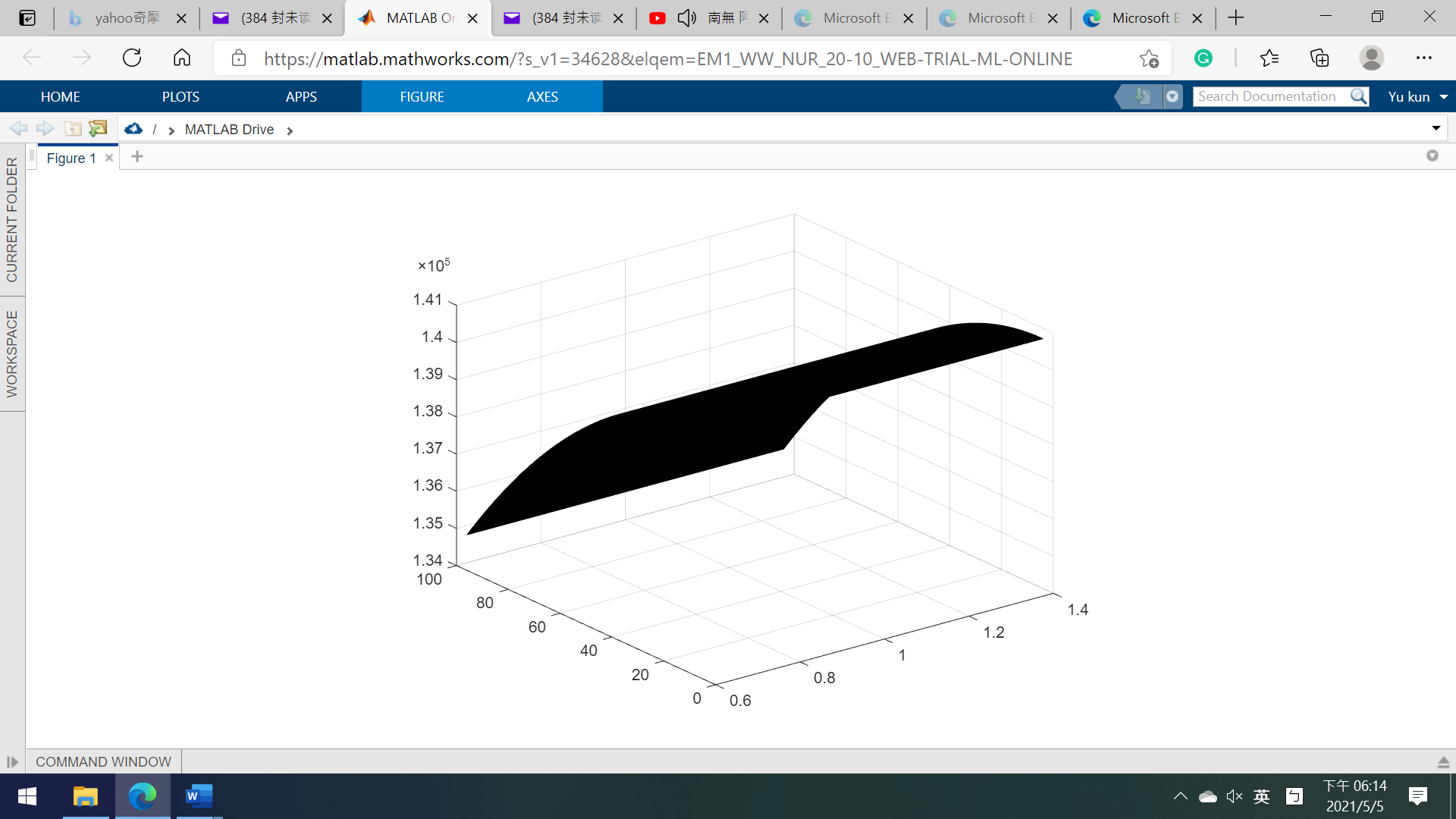


圖二:DTR-GDP,Taiwan,1990-2020

(3)間接稅與GDP的Matlab分析

可知X 對y3= (5.34*exp(-16)*x^3-(1.92*exp(-0.8)*x^2+0.24611*x+369410.2 作一階導數

得

3* 5.34*exp(-16)*x^2-2*1.92*exp(-0.8)*x+0.24611=0

在Mathlab輸入資料如下 可得臨界點x1,x2如下

X1=9.5707e+05

X2=0.1426


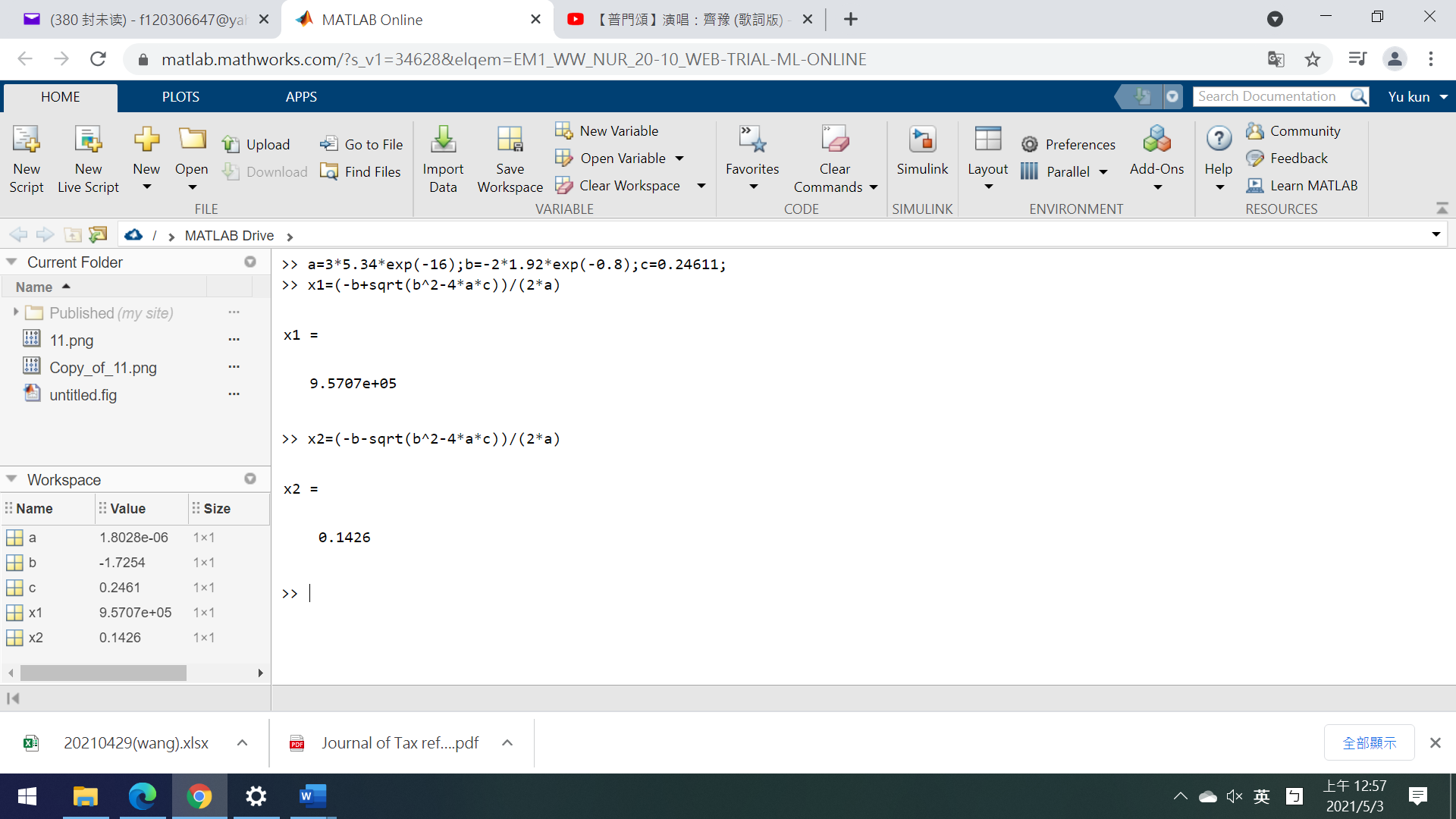


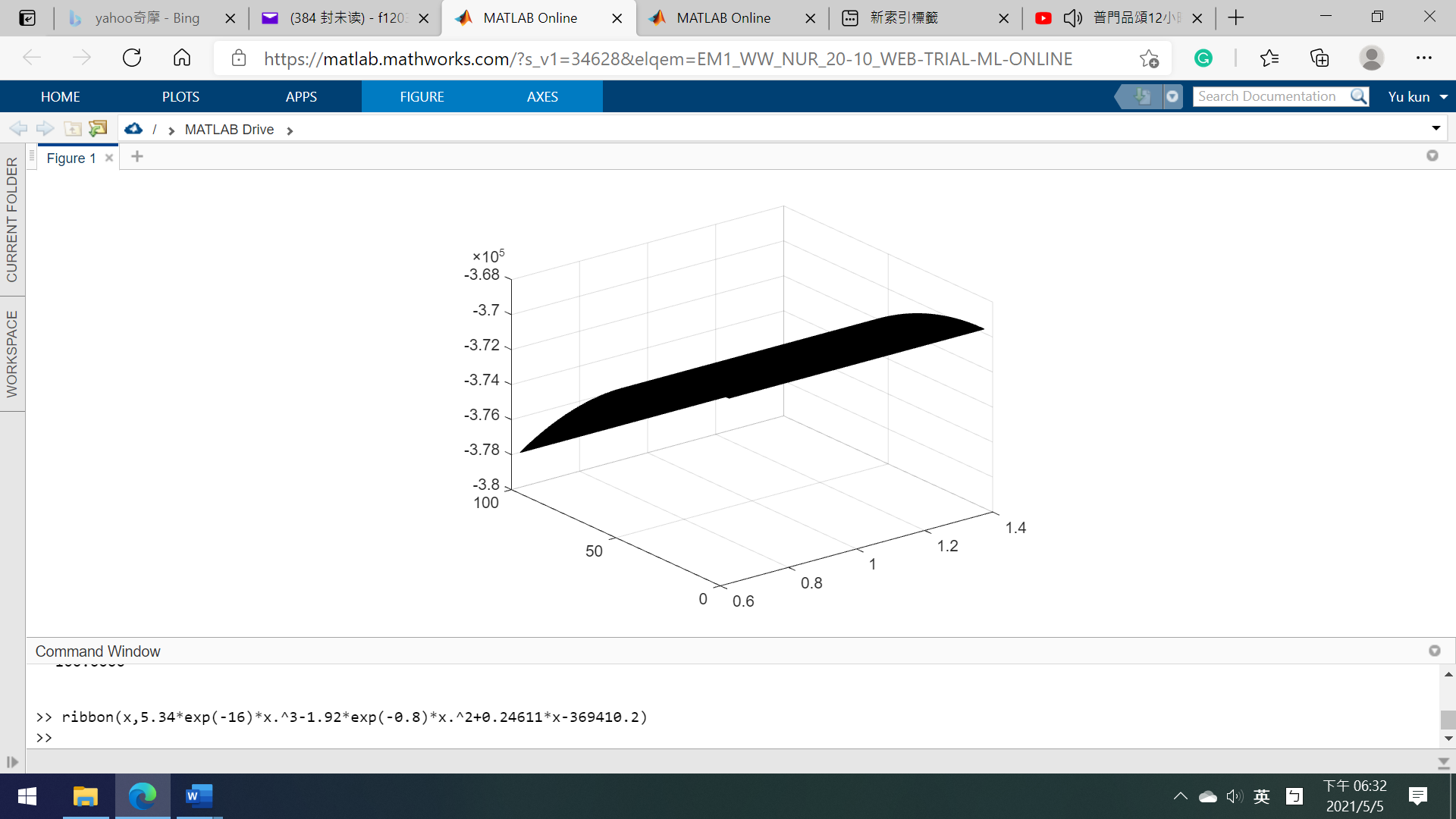


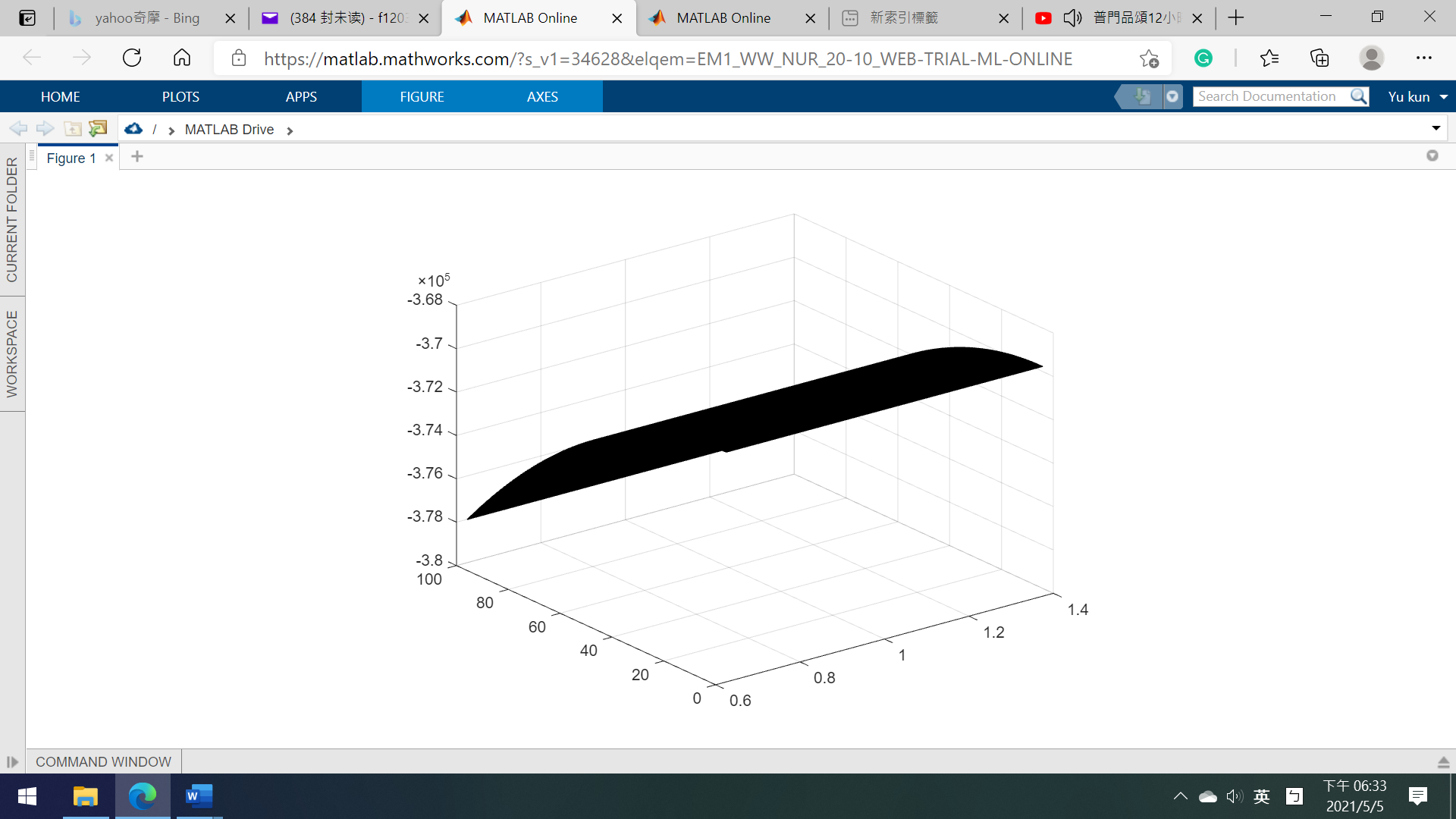


圖三: IDTR-GDP,Taiwan,1990-2020
