# Supplementary material for: Threshold analysis regarding the optimal tax rate and tax evasion. Empirical evidence from Taiwan
Source: PLoS One. 2023 Mar 31;18(3):e0281101. doi: 10.1371/journal.pone.0281101 (PMC10065287; doi:10.1371/journal.pone.0281101)
Supplement: S3 File — (DOCX) [file pone.0281101.s003.docx]

台灣GDP-to-TTR到達拐點了嗎?

一元三次方程在线计算器

表單的頂端

| 例如：输入a=1, b=8, c=16 d=10。 | | | |
| --- | --- | --- | --- |
| ax^3^ + bx^2^ + cx + d = 0 | | | |
| x^3^ + | x^2^ + | x+ |  d = 0 |
|  | | | |

| X1: |  +   i |
| --- | --- |
| X2: |  +   i |
| X3: |  +   i |

表單的底部

[一元三次方程在线计算器 (99cankao.com)](http://www.99cankao.com/algebra/cubic-equation.php)

[一元二次方程式计算器 (520101.com)](https://tool.520101.com/calculator/yiyuanerci/)

由 Case 1

Equation

TTR=c(1)+c(2)*gdp+c(3)*squaregdp+c(4)* triplegdp

TTR=-228482.3+ 0.30713*GDP-2.07E-08*SQUAREGDP+6.26E-16*TRIPLEGDP

取F.O.C 條件

3*6.26E-16squaregdp-2*2.07E-08gdp+0.30713=0

18.78E-16squaregdp-4.14E-08gdp+0.30713=0

可知 GDP= 11,022,364

也就是當GDP= 11,022,364 ,總稅收TTR最大(MTR拐點)

將19766240 代入

TTR=-228482.3+ 0.30713*GDP-2.07E-08*SQUAREGDP+6.26E-16*TRIPLEGDP

TTR=-228482+6070805-8087577+4834443

可得 TTR=2,589,189(Million TWD)

但台灣官方數據顯示2020年的GDP為

19,766,240(Million TWD),TTR為2,398,667(Million TWD)

可知台灣2020年GDP已超過稅收最大值拐點KC, 表示當GDP超過11,022,364, 隨著GDP提高 TTR不增反減，有稅基侵蝕，稅基侵蝕金額為190522(Million TWD)，稅基侵蝕率為0.009638

(=190522/19766240)

台灣GDP-to-DTR到達拐點了嗎?

表單的底部

由 Case 4

Equation

DTR=c(1)+c(2)*gdp+c(3)*squaregdp+c(4)* triplegdp

|  | Coefficient | Std. Error | t-Statistic | Prob. |
| --- | --- | --- | --- | --- |
|  |  |  |  |  |
|  |  |  |  |  |
| C(1) | 140925.6 | 372868.6 | 0.377950 | 0.7085 |
| C(2) | 0.061019 | 0.103792 | 0.587900 | 0.5617 |
| C(3) | -1.52E-09 | 8.91E-09 | -0.170063 | 0.8663 |
| C(4) | 9.13E-17 | 2.39E-16 | 0.381808 | 0.7057 |

DTR=140925.6+ 0.061019*GDP-1.52E-09*SQUAREGDP+9.13E-17*TRIPLEGDP

取F.O.C 條件

3*9.13E-17squaregdp-2*1.52E-09gdp+0.061019=0

27.39E-17squaregdp-3.04E-08gdp+0.061019=0

[一元二次方程式计算器 (520101.com)](https://tool.520101.com/calculator/yiyuanerci/)

GDP=108,944,533(Million TWD)

GDP=2,044,878(Million TWD)

可知 GDP= 2044878, GDP=108,944,533(Million TWD) ,總稅收TTR最大(MTR拐點)

將2020年官方的GDP19,766,240(Million TWD)

代入上式

DTR=140925.6+ 0.061019*GDP-1.52E-09*SQUAREGDP+9.13E-17*TRIPLEGDP

可知 DTR=1,458,258(Million TWD)

但2020年官方實際的數據DTR為1,324,208(Million TWD)

顯示2020年的DTR稅基侵蝕金額為134,050(Million TWD)

DTR的稅基侵蝕率為0.006781 (=134050/19766240)

TTR=c(1)+c(2)*gdp+c(3)*squaregdp+c(4)* triplegdp

f(x)=-228482.3+0.30713x-0.0000000207x²+0.000000000000000626x³

Official Data

A(GDP=19766240, TTR=2398667)

Kuznet Curve approach

B(GDP=19766240, TTR*=2589189)

C=Extremum(f)

D=Inflection(f)


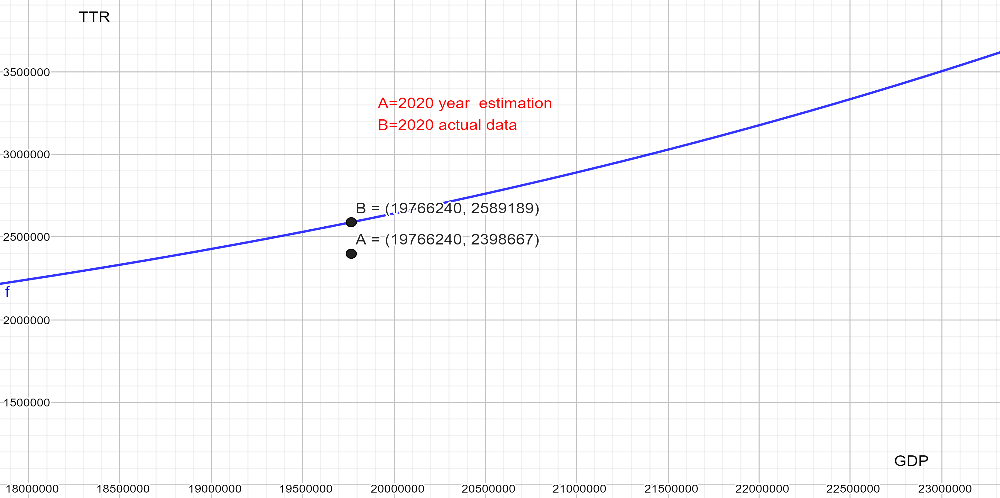


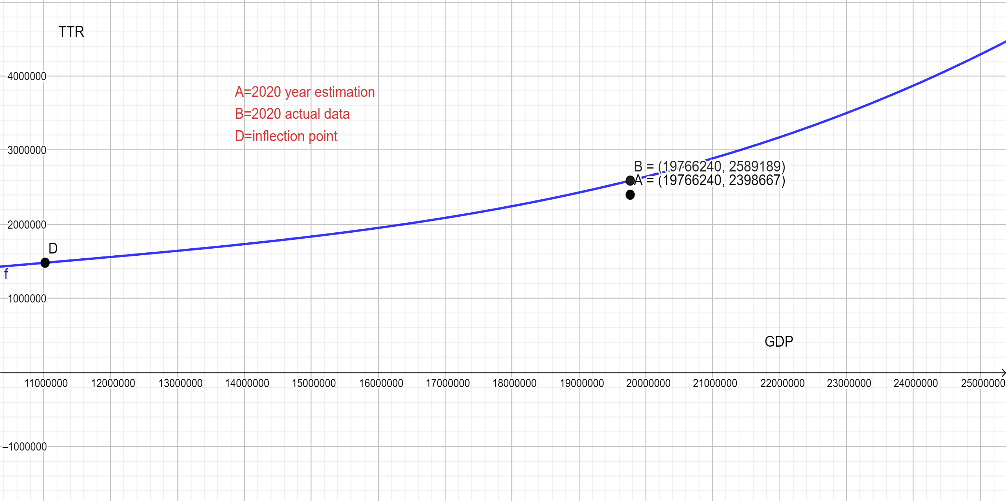


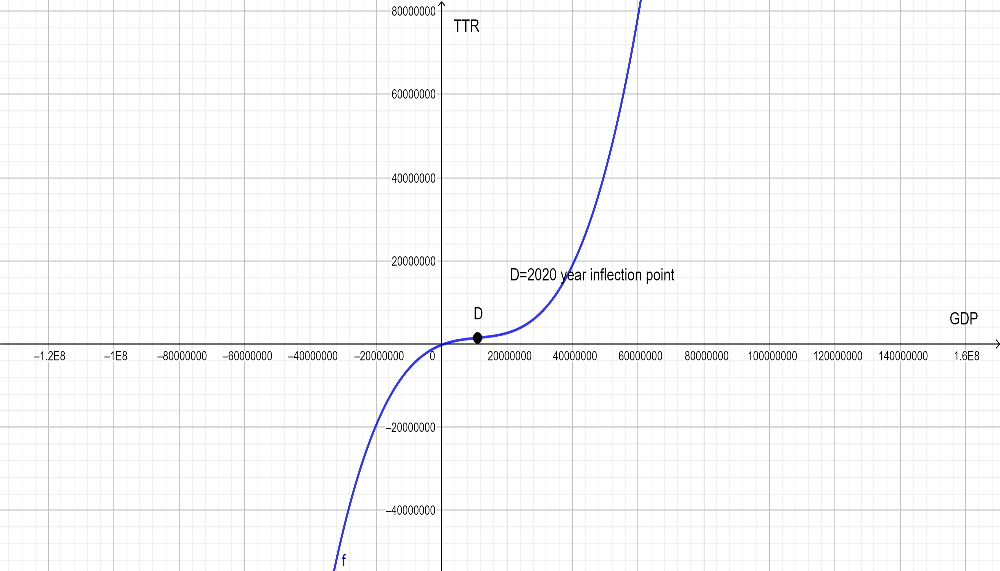


DTR=140925.6+ 0.061019*GDP-1.52E-09*SQUAREGDP+9.13E-17*TRIPLEGDP

f(x)=140925.6+0.061019x-0.00000000152x²+0.0000000000000000913x³

Official Data

A(GDP=19766240, DTR=1324208)

Kuznet Curve approach

B(GDP=19766240, DTR*=1,458,258)

C=Extremum(f)

D=Inflection(f)


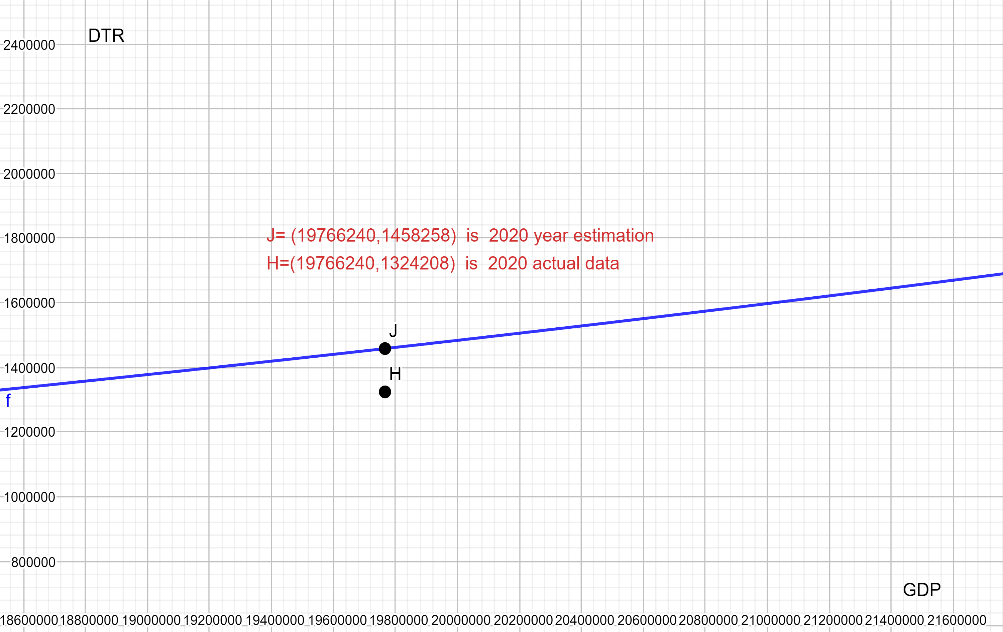


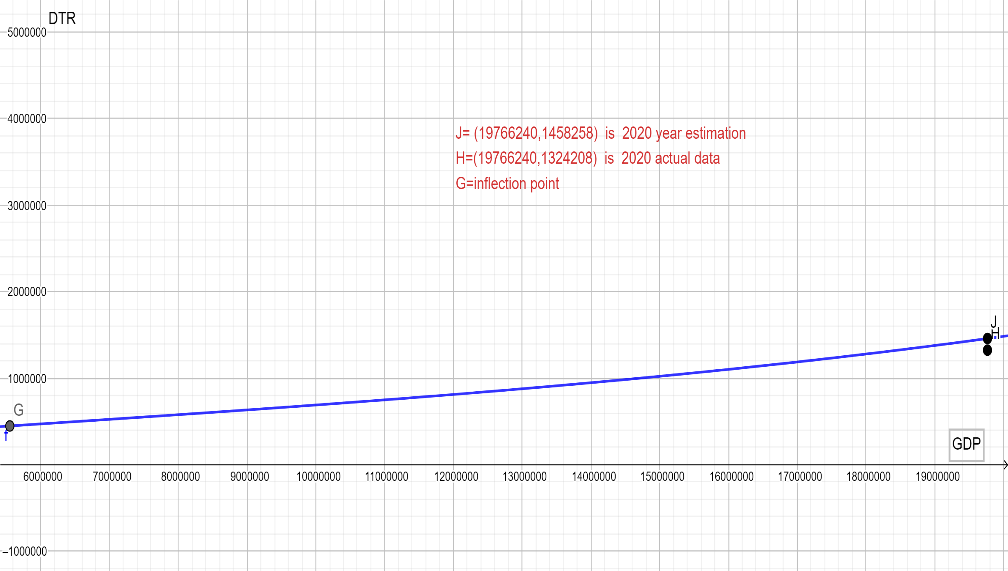


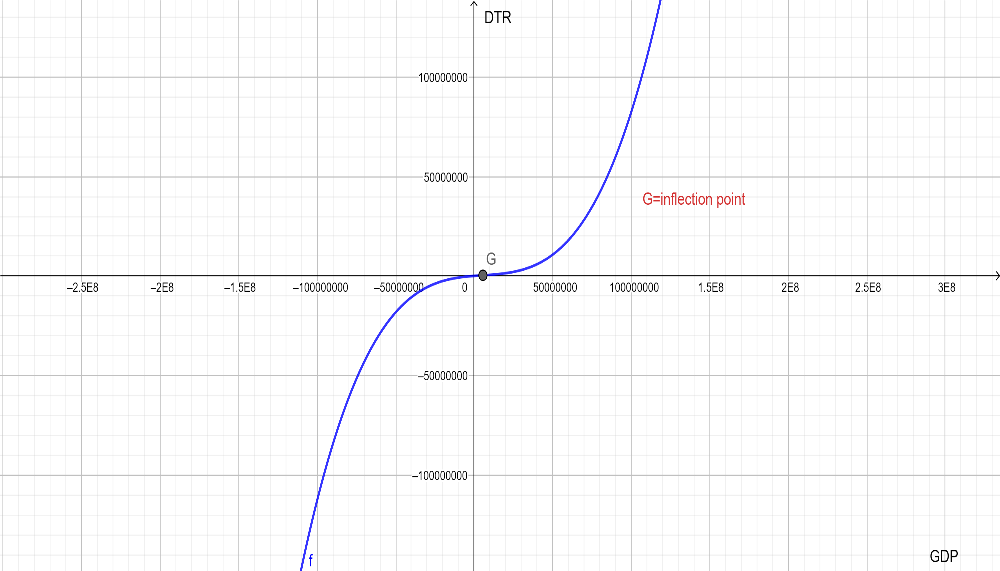


台灣GDP-to-ITR到達拐點了嗎?

表單的底部

Case 1

nondirecttaxrevenue=c(1)+c(2)*gdp+c(3)*squaregdp+c(4)* triplegdp

| System: UNTITLED | | |  |  |
| --- | --- | --- | --- | --- |
| Estimation Method: Seemingly Unrelated Regression | | | | |
| Date: 04/29/21 Time: 00:13 | | | |  |
| Sample: 1991 2020 | | |  |  |
| Included observations: 30 | | | |  |
| Total system (balanced) observations 30 | | | | |
| Linear estimation after one-step weighting matrix | | | | |
|  |  |  |  |  |
|  |  |  |  |  |
|  | Coefficient | Std. Error | t-Statistic | Prob. |
|  |  |  |  |  |
|  |  |  |  |  |
| C(1) | -369410.2 | 291313.4 | -1.268085 | 0.2160 |
| C(2) | 0.246111 | 0.081090 | 3.035027 | 0.0054 |
| C(3) | -1.92E-08 | 6.96E-09 | -2.759365 | 0.0105 |
| C(4) | 5.34E-16 | 1.87E-16 | 2.862245 | 0.0082 |
|  |  |  |  |  |
|  |  |  |  |  |
| Determinant residual covariance | | 4.24E+09 |  |  |
|  |  |  |  |  |
|  |  |  |  |  |
|  |  |  |  |  |
| Equation: NONDIRECTTAXREVENUE=C(1)+C(2)*GDP+C(3) | | | | |
| *SQUAREGDP+C(4)* TRIPLEGDP | | | | |
| Observations: 30 | | |  |  |
| R-squared | 0.836427 | Mean dependent var | | 754240.8 |
| Adjusted R-squared | 0.817553 | S.D. dependent var | | 163847.2 |
| S.E. of regression | 69985.45 | Sum squared resid | | 1.27E+11 |
| Durbin-Watson stat | 2.257293 |  |  |  |
|  |  |  |  |  |
|  |  |  |  |  |

ITR=-369410.2+ 0.246111*GDP-1.92E-08*SQUAREGDP+5.34E-16*TRIPLEGDP

取F.O.C 條件

16.02*E-16squaregdp-3.84E-08gdp+0.246111=0

[一元二次方程式计算器 (520101.com)](https://tool.520101.com/calculator/yiyuanerci/)

GDP=11985018(Million TWD)

可知 GDP= 11985018(Million TWD) ,總稅收TTR最大(MTR=0)

將2020年官方的GDP19,766,240(Million TWD)

代入上式

ITR=-369410.2+ 0.246111*GDP-1.92E-08*SQUAREGDP+5.34E-16*TRIPLEGDP

=-369410.2+0.246111*19766240-0.0000000192*19766240*19766240

+0.000000000000000534*19766240*19766240*19766240=

-369410.2+4864689-7501521+4123950=1117707

可知 ITR=1117707(Million TWD)

但2020年官方實際的數據ITR為1074459(Million TWD)

顯示2020年的ITR稅基侵蝕金額為43248(Million TWD)

ITR的稅基侵蝕率為0.002187 (=43248/19766240)

ITR=-369410.2+ 0.246111*GDP-1.92E-08*SQUAREGDP+5.34E-16*TRIPLEGDP

f(x)
