# Supplementary material for: Threshold analysis regarding the optimal tax rate and tax evasion. Empirical evidence from Taiwan
Source: PLoS One. 2023 Mar 31;18(3):e0281101. doi: 10.1371/journal.pone.0281101 (PMC10065287; doi:10.1371/journal.pone.0281101)

1.TTR

f(x)= (6.26*exp(-16)*x^3-(2.07*exp(-0.8)*x^2+0.30713*x-228482.3

ribbon(x, 6.26*exp(-16)*x.^3-2.07*exp(-0.8)*x.^2+0.30713*x-228482.3)

title('f(x)=6.26*exp(-16)*x.^3-2.07*exp(-0.8)*x.^2+0.30713*x-228482.3');

xlabel('X'),ylabel('Y'),zlabel('Z')


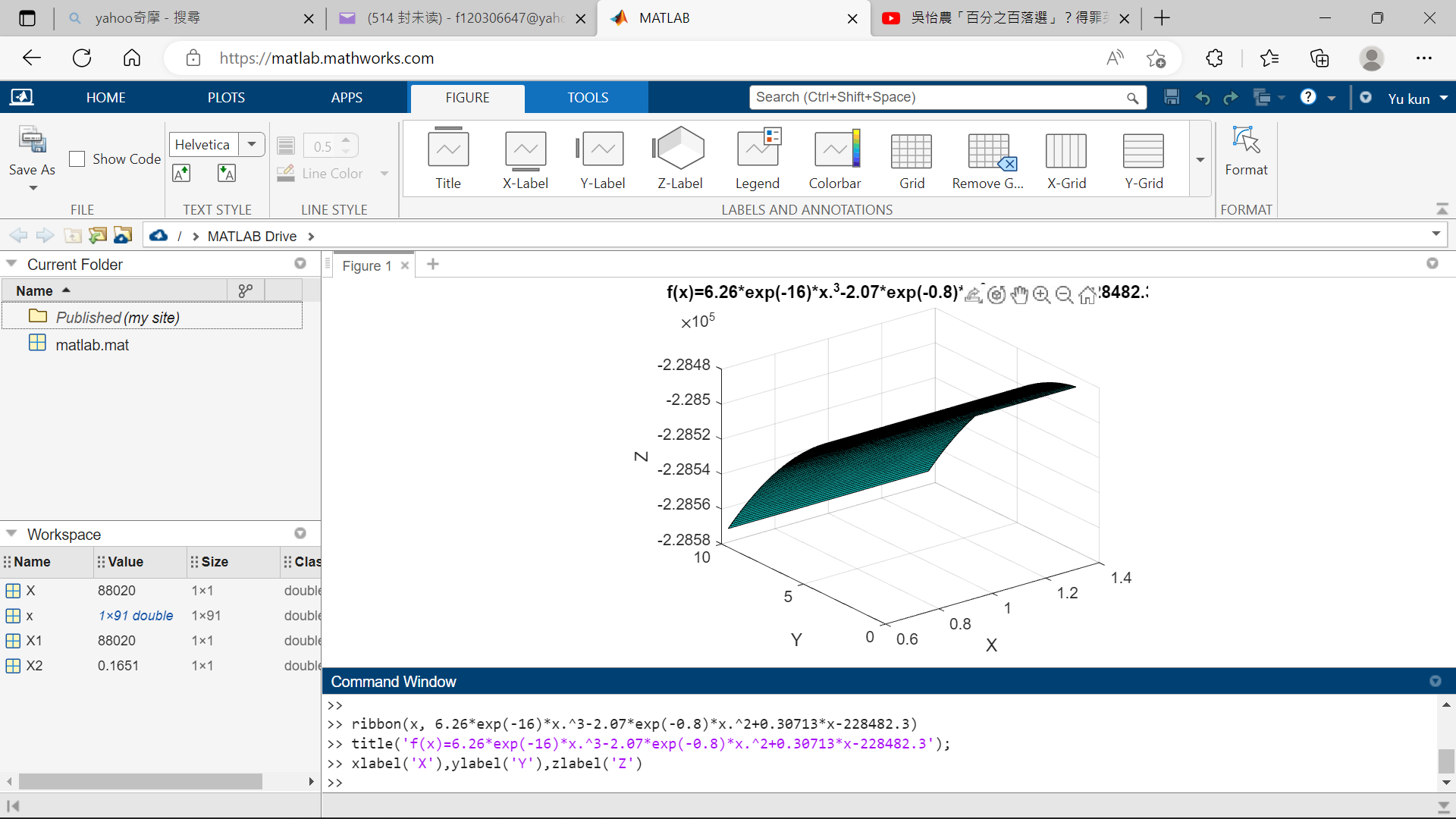


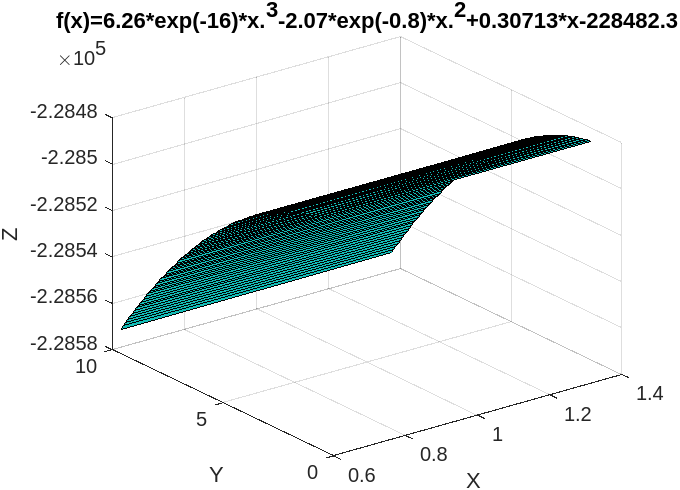


2.DTR

f(x)= 9.13*exp(-17)*x.^3-1.52*exp(-0.9)*x.^2+0.061019*x+140925.6

X=0:0.1:10;

ribbon(x, 140925.6+0.061019*x-1.52*exp(-0.9)*x.^2+ 9.13*exp(-17)*x.^3)

title('f(x)= 9.13*exp(-17)*x.^3-1.52*exp(-0.9)*x.^2+0.0610*x+140925.6');

xlabel('X'),ylabel('Y'),zlabel('Z')


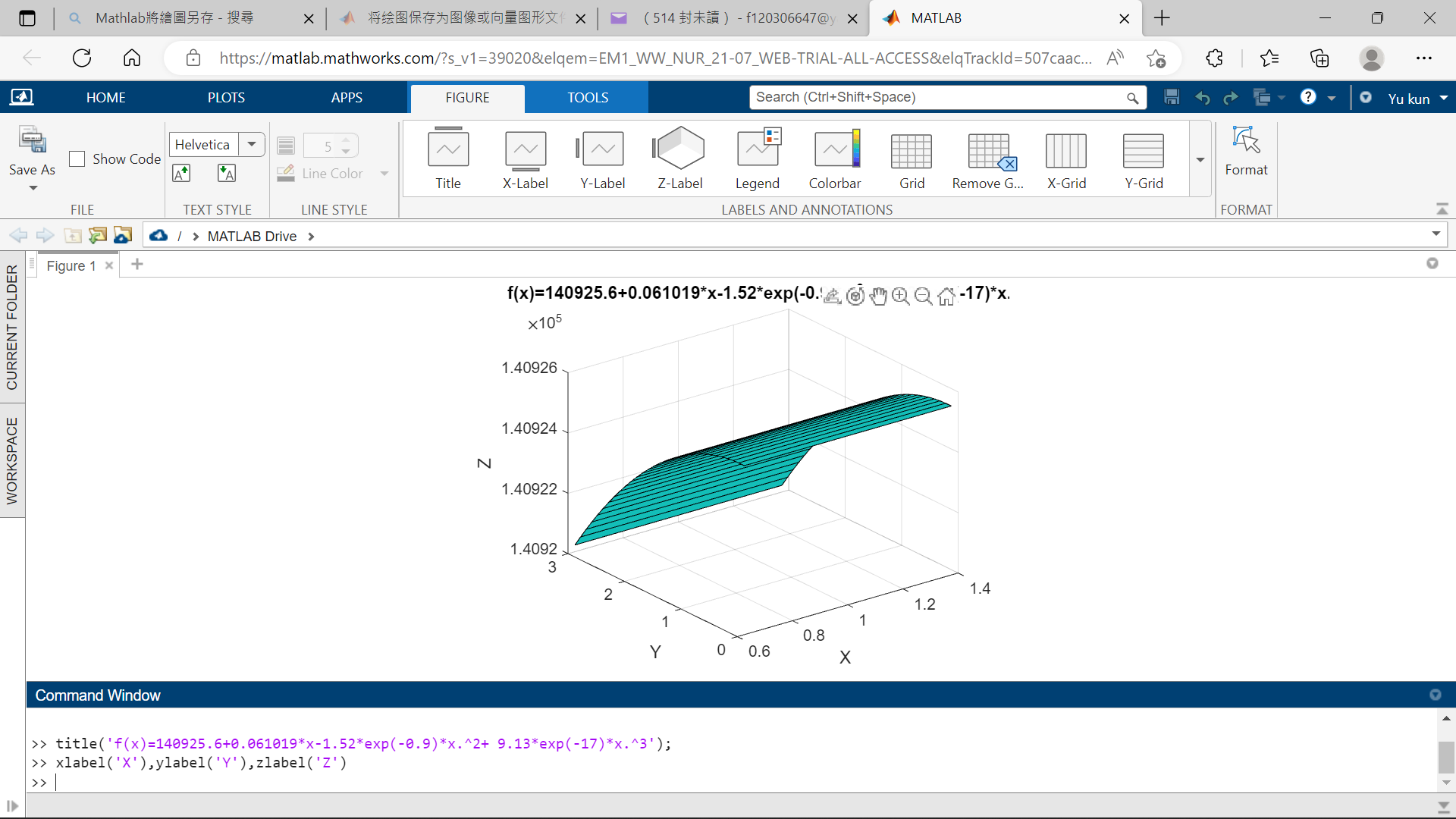


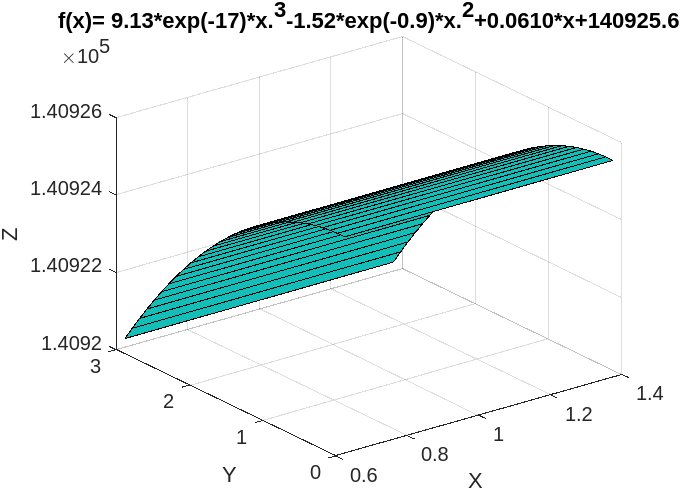


3.ITR

f(x)= 5.34*exp(-16)*x.^3-1.92*exp(-0.8)*x.^2+0.246111*x+369410.2

ribbon(X,369410.2+0.246111*X-1.92*exp(-0.8)*X.^2+5.34*exp(-16)*X.^3)

title('f(x)= 369410+0.24611*x-1.92*exp(-0.8)*x.^2+5.34*exp(-16)*x.^3');

xlabel('X'),ylabel('Y'),zlabel('Z')

X1=95707

X2=0.1426

X1=9.5707e+05

X2=0.1426

X=0:0.1:10;


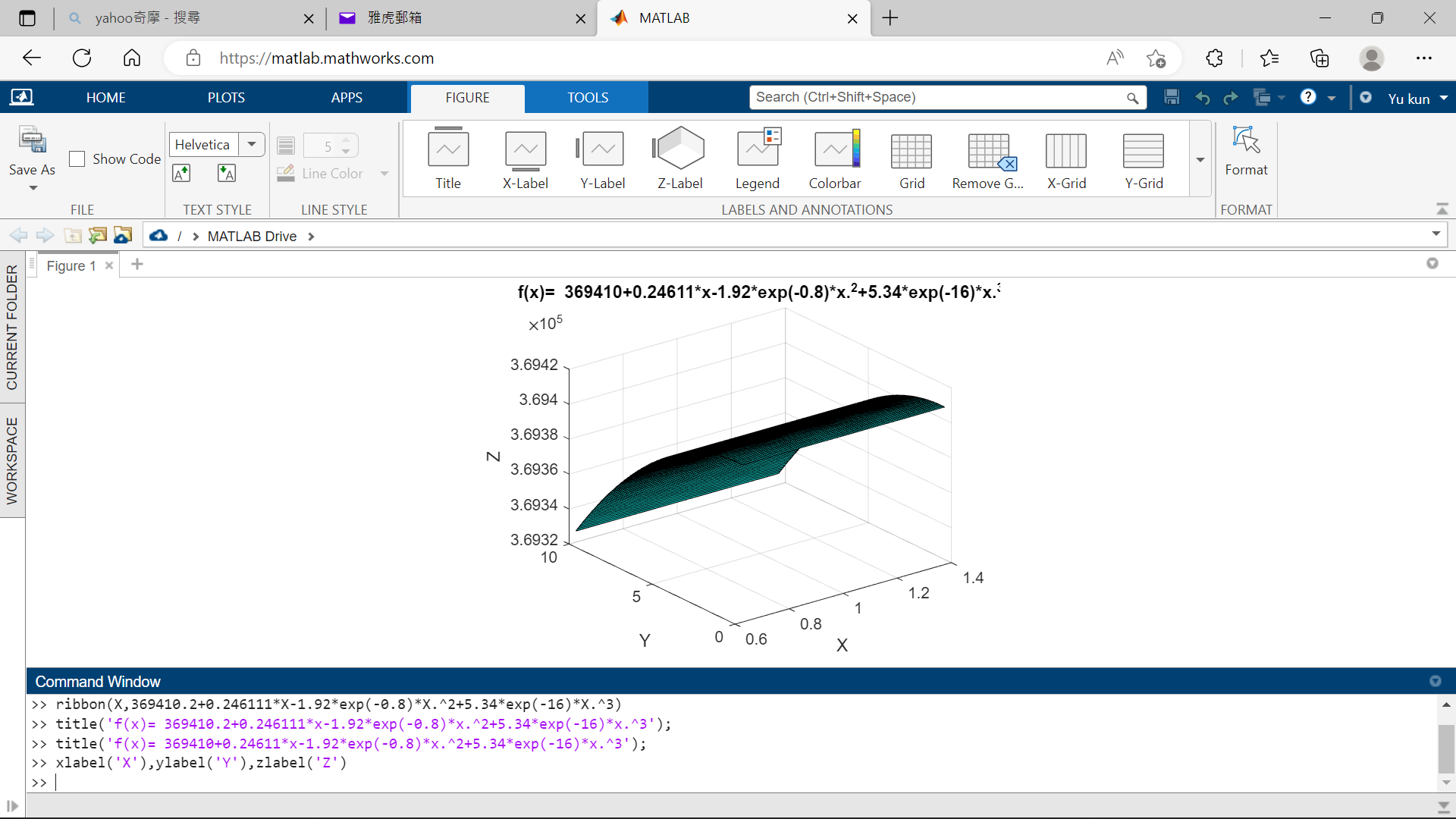


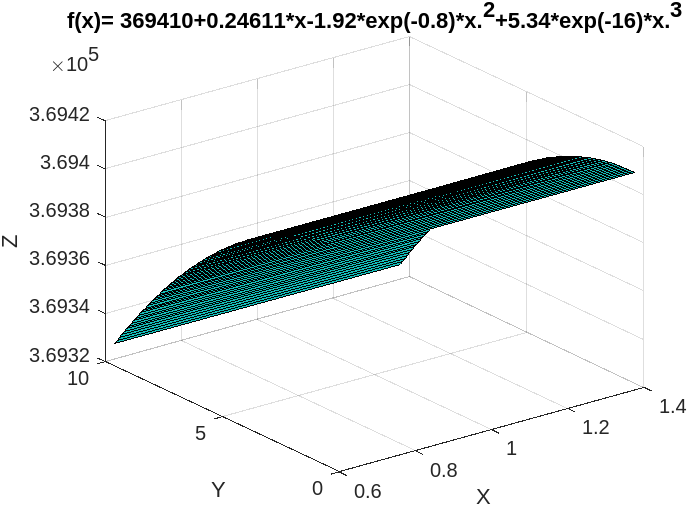

Supplement: S4 File — (DOCX) [file pone.0281101.s004.docx]
