# Supplementary material for: Threshold analysis regarding the optimal tax rate and tax evasion. Empirical evidence from Taiwan
Source: PLoS One. 2023 Mar 31;18(3):e0281101. doi: 10.1371/journal.pone.0281101 (PMC10065287; doi:10.1371/journal.pone.0281101)
Supplement: Attachment — Submitted filename: Review Plos One.pdf [file pone.0281101.s006.pdf]

Review report

of the article:

Optimal tax rate and tax evasion; threshold from Taiwan

I have for the authors the following recommendations for improving the manuscript:

1. I recommend the title modification: Threshold analysis regarding the optimal tax rate and tax evasion. Empirical evidence from Taiwan
2. The introductory part must encompass the research's scope and objectives and the paper's main added value regarding the research questions.
3. The literature review section is missing. There are titles and citations in the introductory part, but the current state of the art must be inserted and expanded. It will be interesting if the authors can introduce a table with the main similar empirical research outcomes both in Taiwan and at the international level.
4. The model part is interesting but must also encompass the research hypothesis. Without any research questions, there is no connectivity between theory and practice.
5. The name and implications must be inserted in the Kuznets Tax Curve Analysis under each subgraph for better and easier identification by the audience.
6. I recommend a discussion and a recommendation section where the authors will debate and analyze the current research paths with the ones that have been debated in the empirical, studies literature.
7. The conclusions section must be expanded with the limitations of the study and with the path for the upcoming future research developments in the field for the authors.
8. The reference section must be seriously improved. The references are quite old and must be updated. The last quoted title is from 2018. There must be updated the literature review section with 10-15 titles from the 2019-2022 period.
