# Supplementary material for: Threshold analysis regarding the optimal tax rate and tax evasion. Empirical evidence from Taiwan
Source: PLoS One. 2023 Mar 31;18(3):e0281101. doi: 10.1371/journal.pone.0281101 (PMC10065287; doi:10.1371/journal.pone.0281101)
Supplement: Attachment — Submitted filename: Response to Reviewers.docx [file pone.0281101.s007.docx]

Please use the space provided to explain your answers to the questions above. You may also include additional comments for the author, including concerns about dual publication, research ethics, or publication ethics. (Please upload your review as an attachment if it exceeds 20,000 characters)

Reviewer #1: Review report of the article:
Optimal tax rate and tax evasion; threshold from Taiwan

I have for the authors the following recommendations for improving the manuscript:

| Reviewer suggestion | Author Response |
| --- | --- |
| 1. I recommend the title modification: Threshold analysis regarding the optimal tax rate and tax evasion. Empirical evidence from Taiwan | Thank you for the valuable comments from the review committee. The title has been revised according to the review committee's suggestion. |
| 2. The introductory part must encompass the research's scope and objectives and the paper's main added value regarding the research questions. | Thank you for the valuable comments from the review committee,  the research scope, objectives and contributions of this paper have  been added to the introduction. |
| 3. The literature review section is missing. There are titles and citations in the introductory part, but the current state of the art must be inserted and expanded. It will be interesting if the authors can introduce a table with the main similar empirical research outcomes both in Taiwan and at the international level. | Yes, our article has listed the relevant literature on threshold theory in other countries and Taiwan, and introduced Taiwan's articles on threshold theory in a separate list |
| 4. The model part is interesting but must also encompass the research hypothesis. Without any research questions, there is no connectivity between theory and practice. | 1. Thank you for your valuable comments, the research hypothesis of this paper has been expressed in 3.1 Methodology and Hypothesis.  2.Our model has encompassed the research hypothesis and connected with the following empirical analysis  3.In section 3.2, based on the theoretical models derived from Eq.(1) to (9). we seek to use empirical analyses to discuss the existence of TTR/DTR/ITR-to-GDP ratio/ Kuznets Curve and further discuss whether the Kuznets Tax Curve/ TTR/DTR/ITR-to-GDP ratio exists in Taiwan covering the 1991-2020. |
| 5. The name and implications must be inserted in the Kuznets Tax Curve Analysis under each subgraph for better and easier identification by the audience. | Thank you so much for your valuable comments,the name and meaning have been inserted under each subgraph in the Kuznets tax curve analysis. |
| 6. I recommend a discussion and a recommendation section where the authors will debate and analyze the current research paths with the ones that have been debated in the empirical, studies literature. | Thank you so much for your valuable comments, the discussion and suggestion section has been added to section 5.2 |
| 7. The conclusions section must be expanded with the limitations of the study and with the path for the upcoming future research developments in the field for the authors. | Yes, we have already added future research direction in Conclusion section. |
| 8. The reference section must be seriously improved. The references are quite old and must be updated. The last quoted title is from 2018. There must be updated the literature review section with 10-15 titles from the 2019-2022 period. | Yes, we have added relevant literature on threshold theory and empirical analysis from 2019 to 2022 in reference section. Thank you so much for your valuable comments. |

Reviewer #2: Manuscript Number: PONE-D-22-29289
Title: Optimal tax rate and tax evasion; threshold analysis from Taiwan

This study explains the relationship among economic growth, tax rate, debt, consumer price index and tax revenue. This study differs from the traditional methodology. Authors adopt SUR-OLS method and Threshold approach to estimate the response of economic growth on total tax revenue, direct tax revenue and indirect tax revenue in Taiwan from 1991- 2020. This paper further discusses the impact of total, direct, and indirect taxes on tax rate volatility.
My detail comments are as following.

| The format of manuscripts is not according to PLOS ONE requirement. Format of Manuscript need a comprehensive revision. Quality of tables and figures is poor. Carefully add table and figure numbers. There are too many subsections better follow PLOS ONE template to revise the paper. | 1.Thank you for the valuable comments.  2.Yes, the content and diagram have been modified according to the requirements of PLOS ONE format. |
| --- | --- |
| Abstract: The section is poorly written. The abstract section should clearly mention the objective, methods, and concise results with sequence. Revise accordingly and add all parts, exclude unnecessary sentences and words in this section. Be specific | Yes, the objective, method and research results have been clearly mentioned in abstract section. Thank you for the valuable comments. |
| Introduction: In the introduction section, research objectives are missing. Clarify the background, objectives, research gaps, and innovations in this section. There are too many citations in this section better add an additional section of literature review and include more advance and relevant literature related to tax rate and tax evasion. | 1.Thank you for the valuable comments. 2.In the introduction section, we have added research objectives, clarifies the background, objectives, research gaps and innovation and added the literature review section, including more advanced and relevant literature related to tax rates and tax evasion. |
| Although methods are advanced and appropriate; however, add the relevance and importance of these methods to justify the application in this study. Further add methods and data section in the manuscript Better add Results and Discussion section: Discuss results in detail and mention the innovative outcome. Further results should be backed with appropriate literature. Results should be further elaborated in detail. | 1.Thank you for the valuable comments.  2.We have added the relevance and importance of these methods to demonstrate their application in our study. Additionally, we have added the “Methods and Data” sections to the manuscript. We further supplement the “Results and Discussion” sections with references to innovative results. Further, our empirical results have been further elaborated. |
| Conclusion: Add limitations and future research ideas in the conclusion section. Expand the policy implications for Taiwan. | Yes, our conclusions section have been expanded with the limitations of the study and with the path for the upcoming future research developments in the field for other authors. |
| References: References are not aligned with Journal Format, Revise it Avoid grammatical and typo errors and revise the manuscripts for these two concerns. | Yes, the References section have been revised according to the format of the journal.In addition, our article has updated the literature review section with 10-15 titles from the 2019-2022 period. |
